# Improving few-shot named entity recognition for large language models using structured dynamic prompting with retrieval augmented generation

**DOI:** 10.1038/s44387-025-00062-2

**Published:** 2026-03-31

**Authors:** Yao Ge, Yuting Guo, Sudeshna Das, Abeed Sarker

**Affiliations:** 1 Department of Biomedical Informatics, School of Medicine, Emory University, Atlanta, GA USA; 2 Department of Computer Science, Emory University, Atlanta, GA USA; 3 National Library of Medicine, National Institutes of Health, Bethesda, MD USA; 4 Department of Biomedical Engineering, Georgia Institute of Technology and Emory University, Atlanta, GA USA

**Keywords:** Computational biology and bioinformatics, Health care, Mathematics and computing

## Abstract

Biomedical named entity recognition (NER) is a high-utility natural language processing task, and large language models (LLMs) show promise in few-shot settings. In this article, we address performance challenges for few-shot biomedical NER by investigating innovative prompting strategies involving retrieval-augmented generation. Using five biomedical NER datasets, we implemented and evaluated a systematically-structured multi-component static prompt and a dynamic prompt engineering technique, where the prompt is dynamically updated via retrieval with most relevant in-context examples based on the input texts. Static prompting with structured components increased average F_1_-scores by 12% for GPT-4, and 11% for GPT-3.5 and LLaMA 3-70B, relative to basic static prompting. Dynamic prompting further boosted performance and was evaluated on GPT-4, LLaMA 3-70B, and the recently released open-weight GPT-OSS-120B model, with TF-IDF based retrieval yielding the best results, improving average F_1_-scores by 8.8% and 6.3% in 5-shot and 10-shot settings, respectively. An ablation study on retrieval pool size demonstrated that strong performance can be achieved with relatively small number of annotated samples, reinforcing the annotation efficiency and scalability of our framework in real-world settings.

## Introduction

Named entity recognition (NER) is a fundamental natural language processing (NLP) task, essential for extracting predefined entities from free text, with wide application in digital medicine. Within the biomedical domain, NER is applied to detect clinical entities such as diseases, adverse drug reactions, treatments, and symptoms, and non-clinical entities such as social determinants of health^1–4^. Despite substantial progress in supervised NER, applying these models to real-world biomedical text remains challenging^5^. The domain is characterized by the sparsity of specific medical concepts, such as rare health conditions, and specialized language that varies considerably across sub-domains like radiology, pathology, and oncology. These variations, along with differing institutional documentation practices, create non-trivial domain shifts^6^. While some deep neural network-based NER methods have achieved human-like performance, they require large amounts of training data, which is often infeasible to create for specialized biomedical problems. Consequently, constructing large, representative annotated datasets that generalize across diseases and institutions is difficult.

A primary obstacle is that high-quality annotation requires domain expertise and is often impractical in scenarios such as rare diseases or emerging conditions (e.g., early-stage COVID-19), where labeled data may be unavailable. Furthermore, NER systems trained on open-domain data rarely transfer to biomedical tasks without re-training via manual annotation. However, manually annotating data for targeted biomedical NLP problems can be expensive, particularly when medical expertise is required, or infeasible (e.g., for sparsely-occurring medical conditions). Institution-specific documentation styles and EHR systems further introduce lexical variations, limiting the generalizability of supervised models across clinical settings. Even when studies invest in annotation, privacy regulations often prevent the public sharing of datasets derived from electronic health records. Few-shot learning (FSL) offers a promising solution to these challenges by enabling NER systems to generalize effectively to new biomedical domains using small numbers of labeled examples^7^. Beyond addressing data scarcity, few-shot NER approaches support adaptability across institutions, specialties, and evolving biomedical terminologies, contexts where traditional re-training or large-scale relabeling is infeasible. Thus, developing generalized few-shot NER models is critical for scalable and equitable deployment of biomedical NLP tools.

Approaches employing large language models (LLMs), such as the GPT and LLaMA series models, have advanced FSL-based NLP by outperforming traditional NLP approaches in zero-shot and few-shot settings^8^, with transformational impact on restricted domains such as biomedical. By leveraging in-context learning, where training examples are provided as part of the prompt, current LLMs can readily adapt to diverse text structures and vocabularies^9^. A major focus of recent biomedical NLP research has been prompt engineering^10,11^, as carefully designed prompts can help align an LLM’s understanding and outputs with task-specific requirements. The typical approach in prompt-driven methods is to optimize predefined *static* prompts for a target task. Static, in this context, refers to the use of the same, consistent prompt for every instance in a dataset during inference. Thus, regardless of the content of the input text, the model applies the same fixed prompt and in-context examples. This rigidity causes high variance depending on how relevant the static examples happen to be to the unlabeled input^12^. Moreover, prompt-based techniques are constrained by context window limits^13,14^. Even with large context window sizes that allow many examples to be embedded, LLM performances have been consistently shown to be degraded when the text within the context window is too long^15^. Thus, incorporating too many labeled examples within a prompt is not necessarily beneficial.

We hypothesize that dynamically choosing in-context examples to embed into a structured prompt will improve biomedical FSL-based NER performance. To this end, we employ retrieval-augmented generation (RAG). RAG enriches the LLM’s context by retrieving information relevant to a specific query prior to generation^16,17^. Guided by similarity measures like cosine similarity^18^, RAG allows the model to access examples tailored to the input text. This approach addresses limitations related to knowledge cutoffs and domain specificity^19^, potentially improving adaptability in specialized applications by incorporating contextually relevant information during inference^20^. In FSL settings, RAG reduces reliance on large annotated datasets by dynamically selecting the most pertinent data while ensuring prompt length remains manageable^21^. Importantly, RAG is not an alternative to structured prompting, such as chain-of-thought (CoT) prompting^22^, but rather a complementary mechanism with potential to improve NLP performance for complex, sparse entities.

Motivated by these observations, we use RAG to retrieve and embed annotated examples most similar to a given unlabeled text into a rigorously-constructed structured prompt. We first construct a detailed, structured prompt tailored for NER, which includes six elements: (i) task description and entity definitions, (ii) dataset description, (iii) high-frequency instances, (iv) background domain knowledge, (v) feedback from error analysis, and (vi) few-shot examples. Following prompt optimization, we evaluate the effectiveness of the static prompting using GPT-3.5^23^, GPT-4^24^, and LLaMA 3^25^, and evaluate dynamic prompting using GPT-4, LLaMA 3, and the recently released GPT-OSS^26^ model across five datasets. For dynamic prompting with RAG, we also systematically evaluate multiple retrieval mechanisms and investigate how prompt design choices and retrieval pool size affect model performance. Our results demonstrate the potential of these techniques to enhance entity detection in biomedical NER.

The primary contributions of this work are summarized below:

- Development of a framework for structured static prompting, incorporating task-relevant instructions, entity definitions, and dataset contextualization to improve few-shot NER.
- Integration and evaluation of RAG; assessing how distinct retrieval mechanisms (TF-IDF^27^, SBERT^28^, ColBERT^29^, and DPR^30^) enhance dynamic prompting by selecting contextually relevant examples.
- Comparative analysis of static and dynamic prompting strategies, benchmarking their effectiveness in few-shot biomedical NER, and offering insights into their strengths across different datasets.
- An ablation analysis of retrieval pool size, quantifying how different amounts of annotated retrieval data influence performance in RAG-based dynamic prompting.

## Results

In this section, we first present the details and performance metrics for the distinct components of our structured prompt. We then present the results of dynamic prompting via RAG for multiple-shot settings and compare different retrieval mechanisms. Finally, we report an ablation study on retrieval pool size.

### Task-specific static prompting

Table 1 presents the F_1_-scores on identical test sets from five NER datasets for distinct structured prompt components. The results demonstrate consistent performance improvements across datasets and the three LLMs when all prompt components are combined. Compared to the baseline prompt (BP), the addition of task-specific components, such as dataset descriptions, high-frequency instances, error analysis, and few-shot examples, led to significant improvements in F_1_-score across all datasets. GPT-4 showed the largest improvements when the full structured prompt was used; average F_1_-score increased by 12.0%, ranging from 6.95% for MIMIC III to 23.7% for Med-Mentions. GPT-3.5 achieved an average F_1_-score increase of 11.4%, with gains ranging from 7.1% for BC5CDR to 22.9% for Med-Mentions. LLaMA 3-70B, which had the lowest performance for the BP, showed an average F_1_-score increase of 11.1%, with its largest improvement observed on the Med-Mentions dataset (21.4%). GPT-4 consistently outperformed GPT-3.5 and LLaMA 3-70B in all configurations, particularly on the BC5CDR and Med-Mentions datasets. GPT-3.5 and LLaMA 3, while achieving slightly lower overall performance, still exhibited performance improvements relative to the baseline. This is evident in datasets such as REDDIT-IMPACTS, where its F_1_-score exhibited significant improvement with the integration of additional components.

**Table 1:** Performance comparison of various prompting strategies across different datasets

	

Red bold values indicate the best overall performance achieved by combining all prompt strategies (“BP + All above”) for each model-dataset pair. Black bold values denote the strongest performance among individual prompt enhancement strategies (excluding the full combination). Green bold values highlight cases where incorporating external domain knowledge (e.g., UMLS) leads to a degradation in performance compared to the basic prompt, illustrating potential negative effects of certain knowledge augmentations.

**Fig. 1:**
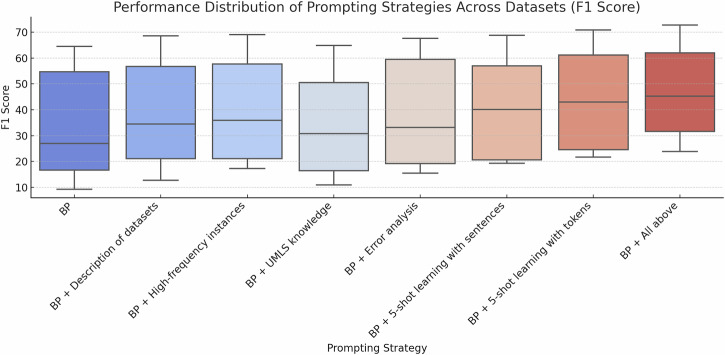
Performance distribution for prompting strategies across datasets (F_1_-score). The box plots depict the performance of various prompting strategies applied to five biomedical datasets, highlighting the range, median, and distribution of F_1_-scores for each strategy.

The box plots in Fig. 1 depict performance changes associated with distinct prompt components across all datasets in the static setting. The incorporation of knowledge from the Unified Medical Language System (UMLS)^31^ improved F_1_-score in certain datasets, such as BC5CDR and Med-Mentions, but underperformed compared to the BP for datasets like REDDIT-IMPACTS and MIMIC III. The intuition behind this component was to provide foundational biomedical knowledge by introducing descriptions and context derived from UMLS. However, this approach proved to be largely ineffective, particularly in datasets that are not strongly aligned with UMLS’s predefined biomedical concepts. For example, in the REDDIT-IMPACTS dataset, GPT-3.5’s F_1_-score decreased slightly from 16.7% to 16.4%, suggesting that the background information from UMLS may have diluted the model’s ability to capture task-specific cues, and instead, degraded performance by increasing the prompt length. Precision and recall results are reported in Table S6 of the Supplementary Materials, and 95% confidence intervals (CIs)^32^ for F_1_-scores are provided in Table S7.

### Dynamic prompting with RAG

The results in Table 2 demonstrate the effectiveness of dynamic prompting in multiple FSL settings (5-shot, 10-shot, and 20-shot) for GPT-4, LLaMA 3, and GPT-OSS across five biomedical datasets. As described in the “Experimental setup” section, the BPs used randomly selected examples, and the results were averaged over four random runs. Detailed results for each random run, along with the averaged results, are presented in Table S1 to Table S5 in Supplementary Materials. Precision and recall results are reported in Table S8 to Table S10 of the Supplementary Materials, while 95% confidence intervals (CIs) for F_1_-score are provided in Table S11 to Table S13. All results are organized and presented separately for each LLM.

**Table 2:** Evaluation of dynamic prompting strategies (5-shot, 10-shot, and 20-shot) using GPT-4, Llama 3 and GPT-OSS across five medical datasets

	

The table presents F_1_-score for each retrieval method: Base Prompt, TF-IDF, SBERT, ColBERT, and DPR. Bold numbers indicate, for each dataset–model–shot configuration (e.g., NCBI with GPT-4 at 5-shot), the retrieval engine with the highest F_1_-score in that column, while red underlined values indicate the overall best performance for that dataset across all models and settings.

All three LLMs benefit significantly from retrieval-based prompt updates, with TF-IDF frequently achieving the highest F_1_-scores across models. On the BC5CDR dataset, TF-IDF achieved the highest F_1_-scores for GPT-4 (85.9%, 86.6%, and 87.2% for 5-, 10-, and 20-shot) and for GPT-OSS (86.0%, 87.8%, and 86.5%, respectively). SBERT and ColBERT both contributed strong gains. Across GPT-4 and LLaMA 3, SBERT generally outperformed ColBERT, whereas for GPT-OSS the two methods performed similarly. As an example, SBERT exhibited strong performance on the REDDIT-IMPACTS dataset, where it achieved the highest F_1_-scores of 33.7% (5-shot) and 35.5% (10-shot) for GPT-4 and 34.4% (5-shot) and 41.4% (20-shot) for LLaMA 3. DPR showed occasional competitive performance, but overall contributed smaller gains compared to others.

**Fig. 2:**
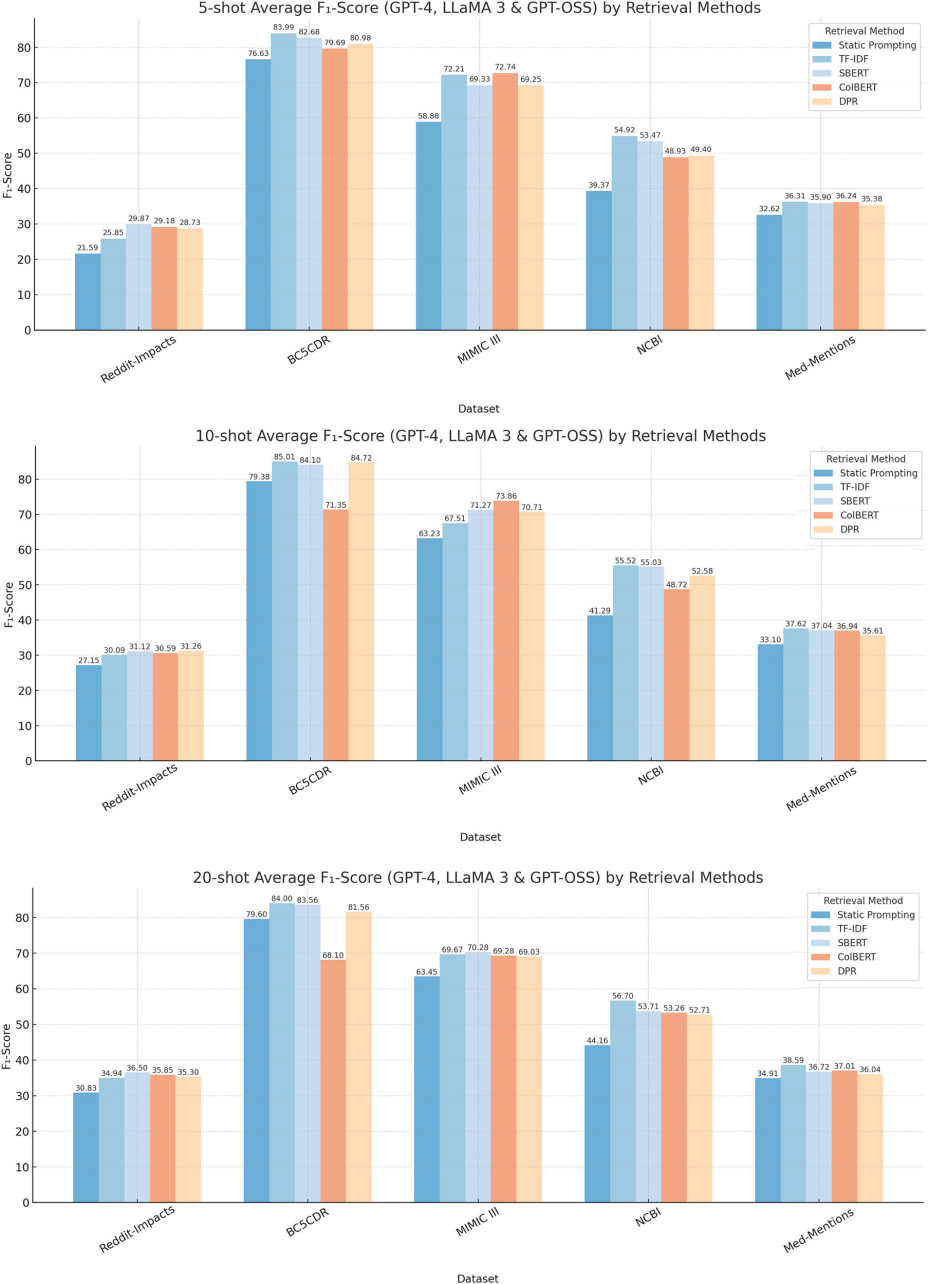
Comparison of average F_1_-scores for GPT-4, LLaMA 3 and GPT-OSS models across different datasets under varying shot settings.

Figure 2 presents the F_1_-scores over the five datasets when using different retrieval methods compared to static prompting for multiple shot settings: 5-shot, 10-shot, and 20-shot. The results are averaged across evaluations conducted using GPT-4, LLaMA 3, and GPT-OSS models. Across all settings, all retrieval-based methods show significant improvements, demonstrating the benefit of incorporating retrieval methods into the prompting strategy.

1. 5-shot Analysis: The SBERT retrieval engine achieved the highest average F_1_-score for the REDDIT-IMPACTS dataset (29.9%), while TF-IDF performed best on the remaining four datasets: BC5CDR (84.0%), MIMIC III (72.2%), NCBI (54.9%), and Med-Mentions (36.3%). TF-IDF achieved an average F_1_-score improvement of 8.8% across all datasets in the 5-shot setting, followed closely by SBERT with 8.4%. ColBERT and DPR showed more modest improvements, with 7.3% and 6.8%, respectively.

2. 10-shot Analysis: In the 10-shot setting, no single retrieval method dominated across all datasets. When averaging over GPT-4, LLaMA 3, and GPT-OSS, DPR achieved the highest F_1_-score on the REDDIT-IMPACTS dataset (31.3%), ColBERT performed best on MIMIC III (73.9%), and TF-IDF obtained the top average performance on BC5CDR (85.0%), NCBI (55.5%), and Med-Mentions (37.6%). Across all model-dataset combinations, SBERT yielded the largest absolute improvement over the base prompt, with an average gain of 6.9%, followed closely by TF-IDF with 6.3% and DPR with 6.1%, while ColBERT provided more modest gains with 3.5%.

3. 20-shot Analysis: TF-IDF demonstrated strong performance, achieving the highest F_1_-scores on three datasets: BC5CDR (84.0%), NCBI (56.7%), and Med-Mentions (38.6%). SBERT performed best on REDDIT-IMPACTS with a top score of 36.5% and also slightly outperformed other methods on MIMIC III with an F_1_-score of 70.3%. Among the retrieval approaches, in the 20-shot setting, TF-IDF achieved the highest average improvement with 6.2%, followed closely by SBERT with 5.6%. DPR showed a moderate improvement with 4.3%, while ColBERT exhibited the lowest increase of 2.1%.

Overall, GPT-4 consistently achieved higher F_1_-scores across all datasets and retrieval methods. In the 5-shot setting, GPT-4 outperformed LLaMA 3 and GPT-OSS by average margins of 3.94% and 5.87%, respectively. This gap widened in the 10-shot setting, where GPT-4 exceeded LLaMA 3 by 5.47% and GPT-OSS by 7.99%. In the 20-shot setting, GPT-4 surpassed LLaMA 3 and GPT-OSS by an average F_1_-score of 8.30% and 7.58%. This improvement was more visible in datasets with sparse or noisy data, where retrieval-augmented methods play a critical role. GPT-OSS showed highly dataset-dependent behavior. It performed competitively and in several cases matched or exceeded GPT-4 on BC5CDR, but lagged behind both GPT-4 and LLaMA 3 on REDDIT-IMPACTS and Med-Mentions. On relatively structured datasets such as NCBI and MIMIC III, GPT-OSS achieved F_1_-scores close to those of LLaMA 3. GPT-OSS scored 1.93% lower in the 5-shot, 2.52% lower in 10-shot, but 0.72% higher in 20-shot. These findings highlight that GPT-4 exhibits the strongest overall generalization, while GPT-OSS excels primarily on cleaner biomedical datasets with well-defined entity structures.

For GPT-4, across all datasets, larger training sizes (20-shot) tend to yield higher F_1_-scores. Specifically, from 5-shot to 10-shot, the mean F_1_-score increased by 2.51%. However, from 10-shot to 20-shot, the performance gains are notably smaller, with F_1_-score increasing by 1.64%. Comparing 5-shot to 20-shot, the models achieved a cumulative improvement of 4.16% in F_1_-score. GPT-OSS improved by 0.39% F_1_-score from 5-shot to 10-shot, and by an additional 2.05% from 10-shot to 20-shot, for a total increase of 2.44% in F_1_-score. For LLaMA 3, the increases are less consistent, with the best performance observed at the 10-shot setting across all datasets except for the REDDIT-IMPACTS dataset. From 10-shot to 20-shot, mean F_1_-score decreased by −1.46%.

### Retrieval pool size ablation

Table 3 presents the ablation results examining the effect of retrieval pool size (the number of annotated instances indexed in the retrieval engine) on dynamic prompting. We report the averaged F_1_-scores across the two best retrieval strategies, TF-IDF and SBERT. Additional metrics are reported in Table S14 of the Supplementary Materials, and 95% confidence intervals (CIs) of F_1_-scores are provided in Table S15.

**Table 3:** Performance metrics from ablation experiments to study the impact of retrieval pool size on dynamic prompting performance using LLaMA 3-70B

	

The “Base” setting uses static prompts with randomly selected in-context examples. We compare this baseline with dynamic prompting that retrieves candidate examples from pools of size 50, 100, 200, and All (all annotated training instances). Red bold values indicate the best performance achieved among specific retrieval pool sizes (50, 100, and 200) for a fixed number of in-context shots, while black bold values correspond to results obtained using the full pool (“All”).

Across all datasets and both shot settings, increasing the retrieval pool size generally leads to consistent performance gains over the static baseline (Base). For example, in the 5-shot setting, expanding the pool from 50 to 200 examples improved F_1_-score from 75.07% to 78.45% on BC5CDR and from 68.81% to 71.51% on MIMIC III, with similar upward trends observed for NCBI, MedMentions, and Reddit-Impacts. While the “All” condition, which uses the entire set of available training examples as the retrieval pool, achieved the highest scores on most datasets, the performances achieved with 100–200 examples fall within the 95% confidence intervals. Similar patterns were observed in the 10-shot setting. For instance, on BC5CDR, the F_1_-score increased from 76.28% with a pool size of 50 to 79.22% with 200 examples, and then only marginally more to 80.87% when using the full pool, representing statistically insignificant improvements. For MIMIC III, the F_1_-score actually drops when retrieving from all training data compared to a pool size of 200, for both shot settings.

## Discussion

NER is one of the most commonly applied biomedical NLP tasks. Although the emergence of LLMs has led to substantial improvements in NER performance, important limitations remain in few-shot settings, particularly characterized by low accuracy and high variance. In this paper, we proposed and validated (i) a structured prompting framework, and (ii) a RAG-based dynamic prompting technique for improving NER performance using LLMs in few-shot settings. The structured static prompt provides a consistent interpretative skeleton that clearly defines the task, output format, and entity extraction rules, establishing a stable baseline across models. However, static prompts lack the flexibility to adapt to the semantic variability and domain-specific patterns present in real-world biomedical texts. Dynamic prompting addresses this limitation by injecting retrieved, input-specific exemplars into the prompt, allowing the model to leverage examples that are more relevant to the unlabeled input text. The methods proposed in this paper, extensively validated on five standardized datasets with differing characteristics, present an important step towards operationalizing automated NER from biomedical texts. Our experiments reveal that structured prompting, which benefits from iterative refinement, and dynamically updating prompts based on input texts can improve NER performance. The prompt structure and few-shot in-context learning strategy can be replicated for other biomedical NLP tasks.

The results of the static prompting framework indicate that progressively enriching the base prompt through structured design leads to clear performance improvements across all models. Further, retrieval-based prompting enhances performance by providing task-relevant contextual examples that more effectively align the prompt with the semantic characteristics of the input text, allowing the model to interpret biomedical entities more accurately than when using randomly selected examples. This approach helps narrow the gap between general pretraining knowledge and the specific requirements of the NER task. Importantly, performance gains depend more on the relevance of the selected examples than on their quantity. Including too many examples may unnecessarily increase prompt length, weaken key instructions, and introduce irrelevant patterns that degrade performance. Therefore, retrieval-augmented few-shot systems should focus on selecting semantically relevant examples while maintaining a clear and concise prompt structure. These findings support the use of dynamic prompting strategies that prioritize high-quality contextual examples while keeping prompt complexity under control.

Findings from our empirical explorations about model and retrieval engine performances may also have additional utilities for the research community. GPT-4 consistently achieved the highest F_1_-score across datasets and configurations, demonstrating its robustness in understanding biomedical information with complex semantic structures. This superiority was particularly evident in challenging datasets such as REDDIT-IMPACTS or Med-Mentions, where informal expressions and ambiguous entity mentions are common. In these scenarios, GPT-4 outperformed both LLaMA 3 and GPT-OSS, indicating better capacity to generalize under noisy and heterogeneous conditions.

GPT-OSS exhibited more dataset-dependent variations. While its performance generally lagged behind GPT-4, it demonstrated competitive results on datasets with better structure such as BC5CDR and MIMIC III, where entity boundaries and terminology are more standardized. This suggests that GPT-OSS benefits more strongly from clearly defined biomedical contexts and structured lexical patterns, while it struggles in settings requiring more flexible semantic interpretation, such as REDDIT-IMPACTS. Overall, GPT-OSS performance is comparable to LLaMA 3 on structured corpora, but remains consistently below GPT-4 across the evaluated configurations.

In terms of retrieval methods, a relatively surprising finding is the strong performance of TF-IDF-based retrieval. Despite its simplicity, it outperforms or performs on par with more advanced approaches. Retrieval approaches employing more advanced text representations, such as SBERT, tend to perform better on datasets with high lexical diversity, such as the REDDIT-IMPACTS dataset. This can be attributed to the fact that the semantic embeddings used by this model capture vector-level similarities and relationships between words and phrases, even when they are lexically diverse. Advanced retrieval methods like ColBERT and DPR generally underperformed compared to TF-IDF and SBERT. This may be due to several reasons. ColBERT and DPR rely on dense representations, which, while powerful for general-purpose semantic matching, may fail to capture the precise, domain-specific distinctions critical in biomedical datasets. Furthermore, their reliance on dense embeddings may overfit to irrelevant semantic similarities, retrieving documents that are semantically related but not contextually relevant to the query. Given these findings, our results suggest that TF-IDF is the most efficient option for retrieval in datasets with low lexical diversity, while SBERT is better suited for handling linguistically diverse data. ColBERT and DPR, despite their strengths in general-purpose retrieval, do not provide substantial advantages in this domain and may introduce unnecessary computational overhead.

The effect of shot size on performance is not uniform, as observed in the results across datasets. While increasing the shot size from 5 to 20 generally improves F_1_-scores, the extent of improvement is dataset-dependent. Datasets with formal texts, like BC5CDR, which already benefit from the inclusion of the retrieval engine, exhibit marginal gains with additional examples. In contrast, noisy datasets like REDDIT-IMPACTS are more sensitive to shot size, as more examples help the model adapt to diverse linguistic patterns and reduce misclassifications. 20-shot does not always yield the best results. One reason is diminishing returns: as the number of examples increases, redundancy or noise may be introduced, especially in datasets where retrieval engines already provide strong task-specific context. Another potential reason arises from the inherent constraints of LLMs, such as prompt capacity and information crowding. As the shot size grows, the available space for processing task-specific context diminishes, potentially diluting the effectiveness of the prompt or truncating important information.

Our findings suggest that for NER tasks involving sparsely-occurring entities, RAG-based dynamic prompting is likely to obtain better performance compared to optimized, static prompts. For retrieval, TF-IDF and SBERT consistently performed well. As the number of training instances increased, the impact of dynamic prompting over static prompting (Base) became less visible. This is expected since in high-shot settings, random draws of training instances are likely to contain considerable diversity to enable model generalization. It is also possible that as the number of examples provided for in-context learning increases, the overall increase in the length of the prompt diminishes the performance of the LLMs. The influence of input text length and LLM performance is an area of active research^33^.

The ablation experiment results offer practical guidance for employing our retrieval-augmented dynamic prompting architecture for NER in low-data settings. As expected, the performance of the approach generally increased as the pool of training instances indexed in the database increased. However, a pool size of 100 consistently achieved comparable performance to using the whole training set. In some cases, smaller pools even led to better results. This may be because enlarging the candidate set introduces a mixture of relevant examples and noisy ones, and retrieval models can be sensitive to this balance. These effects are expected and limited in scope, and they do not change the overall trend that moderate pool sizes consistently capture most of the performance benefits of dynamic prompting. Crucially, these moderate pool sizes already recover most of the performance gains associated with indexing the entire annotated training set.

In practice, this means that users deploying NER for a new biomedical entity type do not need to build a large annotated dataset; instead, a small retrieval pool can enable strong performance when combined with a powerful LLM and a structured prompting framework. This reveals a favorable and practically meaningful accuracy-annotation trade-off, and highlights an important direction for future work: identifying which characteristics of small retrieval subsets, such as entity coverage, text diversity, or domain specificity, most strongly influence downstream performance in dynamic prompting.

## Methods

### Static prompt engineering

**Fig. 3:**
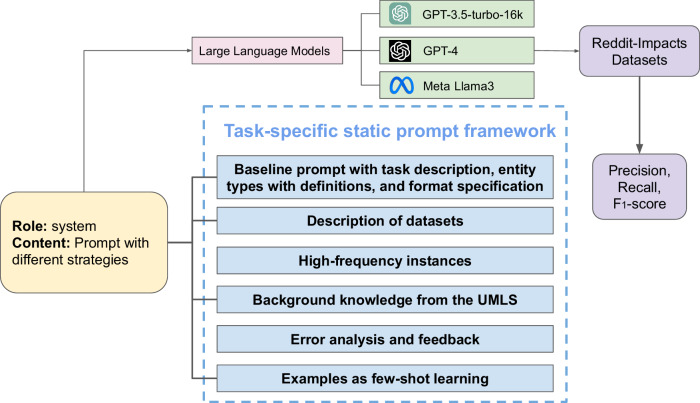
An overview of the NER strategy based on static prompting on three LLMs. Static prompts containing diverse information are provided to the LLMs, which, in turn, generate predictions for evaluation.

Figure 3 presents the components of the static prompt we optimized for the LLMs. We systematically designed task-specific static prompts comprising the following components:

1. Baseline prompt with task description, entity types with definitions, and format specification: The baseline component provides the LLM with essential information regarding the primary aims of the task, which is extracting and classifying entities. The categories of labels present in the dataset, along with their definitions. Entity definitions provide detailed and unequivocal explanations of an entity in the context of a specific task, crucially guiding the LLM toward accurately pinpointing entities within texts. Also, we provided the input, and instructions regarding the output format in the base prompt. For generative LLMs, NER presents greater challenges, relative to classification, as it is essentially a sequence-to-sequence problem, where each token is assigned a corresponding label. However, when a prompt includes a sentence as is, we found that LLMs may struggle to accurately assign labels to each token, resulting in mismatches in the number of input tokens (as annotated in the dataset) and output tokens. This issue is exacerbated by the fact that LLMs have their own tokenization mechanisms, which may differ from the tokenization in the annotated data. If the input and labels are provided in the BIO format instead, it often results in degraded performance due to the LLM’s inability to fully understand the text.

One input approach is to provide a text and indicate the entities within it^34^. For example, in the sentence ‘I was a codeine addict,’ the phrase ‘codeine addict’ is identified as an entity and is annotated as ‘Clinical Impacts’. However, this format can become ambiguous when faced with long sentences that contain the same word or phrase multiple times, each with different contextual meanings, not all of which may be labeled as the relevant entity. Another input method involves providing spans corresponding to the entities^35^, but this also causes mismatches between spans and entities frequently when generative LLMs are used.

To address these challenges, we adopt a new format for constructing the input and output for the LLMs. We provide LLMs with a list of tokens that have already been tokenized. For the output, we instruct the model to return each token, concatenated with its corresponding label. This method allows us to easily extract labels for evaluation, and it ensures a one-to-one correspondence between the predicted labels and tokens, with the number of labels always consistent with the number of tokens in the input sentence.

For example:

Input: [‘I’, ‘was’, ‘a’, ‘codeine’, ‘addict.’]

Output: [‘I-O’, ‘was-O’, ‘a-O’, ‘codeine-B-Clinical_Impacts’, ‘addict.I-Clinical_Impacts’]

To minimize the potential loss of sentence context caused using only tokens, we also explored the effectiveness of using the untokenized sentences as input, and tagged tokens as output:

Input: [‘I was a codeine addict.’]

Output: [‘I-O’, ‘was-O’, ‘a-O’, ‘codeine-B-Clinical_Impacts’, ‘addict.I-Clinical_Impacts’]

2. Description of datasets: By describing a dataset’s origin, content, and themes, we aim to provide LLMs with a basic understanding of the dataset. For example, for the REDDIT-IMPACTS dataset, we described that it focuses on individuals who use opioids, and we are interested in the impact of opioid use on their health and lives.

3. High-frequency instances: Some entities do not have clear definitions, and the determination is more ambiguous. Therefore, we provide the most frequently occurring words or phrases in each entity type within the training dataset to assist LLMs in understanding the potential distribution of entities and the theme of the text for this task. Specifically, we selected high-frequency instances for each class by computing word frequencies from lexicon-annotated data, ranking them by occurrence, and choosing the top 6 high-frequency words as label words for each class. This approach ensures that the selected label words effectively reflect the data distribution and help the model predict appropriate class labels at entity positions. By adding high-frequency instances, we tried to provide the LLM with a lexicon of the concepts of interest.

4. Incorporation of background knowledge from the UMLS: We provide LLMs with comprehensive and structured information we obtained from the UMLS. Our intuition, based on the findings reported in prior work, was that this knowledge could enhance the understanding and interpretation of biomedical concepts, relationships, and terminologies.

5. Error analysis and feedback: To improve the model’s accuracy and address prediction errors, we provide an error analysis and feedback mechanism. After an initial set of predictions was made by LLMs on unseen training set instances, we manually reviewed the errors by comparing the model’s predictions with the gold standard annotations. For each incorrect prediction, we analyze the type and cause of the error, such as misclassification, missed entities, or spurious entities. Based on this analysis, we provide a summarization of feedback to the model. This feedback includes only general descriptions of errors without any examples. While this element of the prompt requires preliminary explorations of the dataset, common possible errors can be identified easily using a small set of training examples, and this enables a mechanism of incorporating expert feedback into the process.

6. Annotated samples: We provide *k* annotated instances within the prompt for in-context learning. Samples are randomly selected and formatted according to the task description and entity markup guide.

We compared the effectiveness of different components of static prompting by incrementally incorporating descriptions of datasets, high-frequency instances, background knowledge from the UMLS, error analysis and feedback, and varying k-shot annotated samples. Detailed prompts used for each dataset are provided in Table S16 to Table S20 in Supplementary Materials.

### Dynamic prompt engineering

In prompt-based strategies using LLMs for in-context learning, the common approach has been to provide the model with a static prompt to guide its predictions. These prompts often include example instances, and CoT prompting. However, a significant limitation of this approach is that the provided examples may differ substantially from the texts from which the model is expected to extract named entities. Note that even in the presence of additional annotated samples, the LLM’s context window size may limit the number of instances that can be embedded in a prompt for in-context learning. A static prompt, thus, does not generalize well, leading to high variance in performance.

**Fig. 4:**
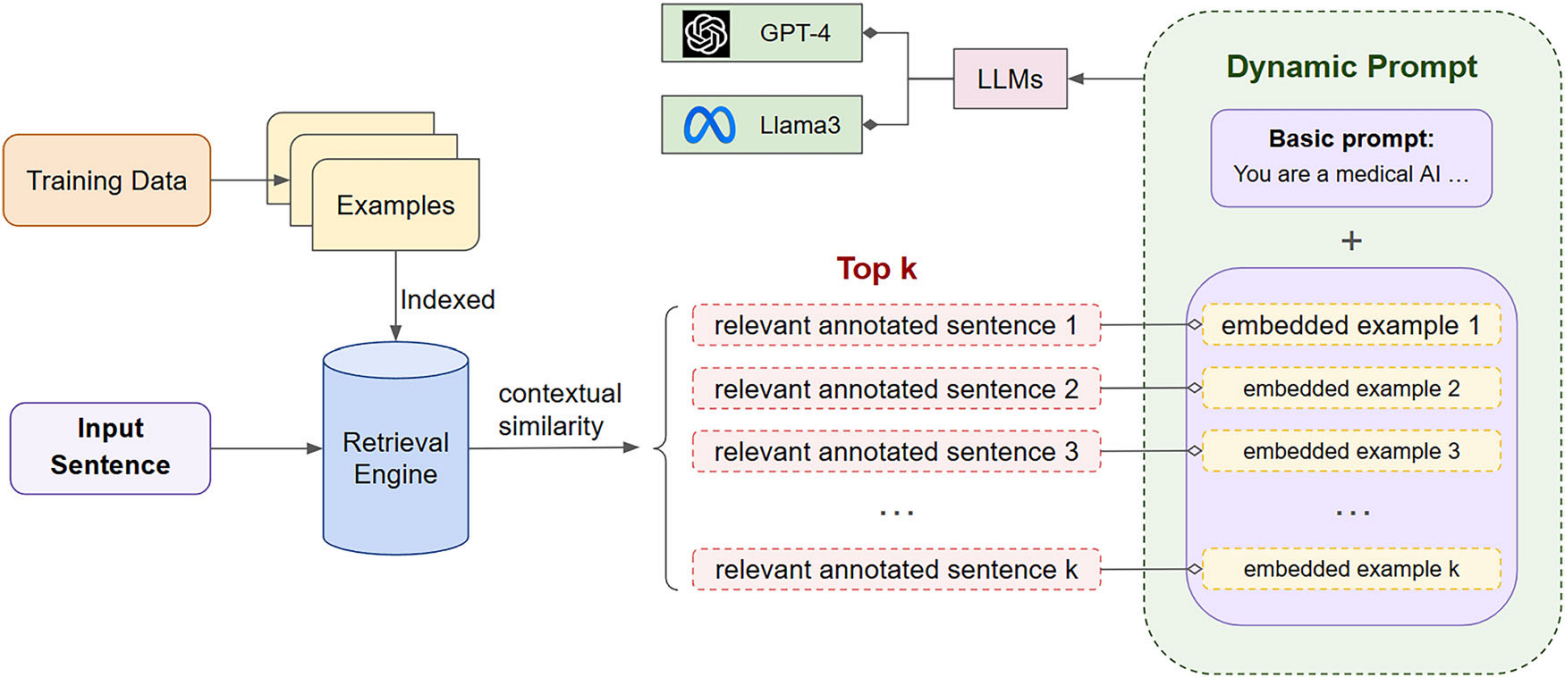
Overview of retrieval-based dynamic prompting model. In the first step, the training data are provided to the retrieval engine for indexing. During inference, the system first ranks all training examples based on contextual similarity with the input text. Finally, the top *n* retrieved instances are embedded in the prompt, which is passed to the LLMs.

To address this issue, we attempted to improve upon static prompting and adopted a dynamic approach involving RAG. In our proposed approach, a retrieval engine is first indexed with the annotated examples from the training set. Upon receiving an input sentence, the system first retrieves the top *n* annotated examples using the retrieval engine. The retrieved examples are then embedded into the prompt, which is then passed to the LLM along with the input text. Figure 4 presents an overview of the system architecture.

Selecting an effective retrieval engine is crucial since the examples embedded in the prompt influence the model’s performance. We considered several retrieval methods, each chosen for its unique strengths in handling diverse biomedical texts, and applicability in FSL settings. The engines we selected are: TF-IDF, Sentence-BERT (SBERT), ColBERT, and Dense Passage Retrieval (DPR). These search mechanisms offer a range of capabilities, from efficient keyword matching to advanced deep-learning-based retrieval. We provide further details below.

1. TF-IDF: Term Frequency-Inverse Document Frequency (TF-IDF) scores the relevance of documents based on the frequency of terms. We included TF-IDF due to its efficiency and simplicity, which allows for rapid retrieval of relevant examples based on keyword overlap. While it lacks semantic understanding, it serves as a strong baseline, particularly when the input contains well-defined biomedical terminologies.
2. Sentence-BERT (SBERT): SBERT leverages a pre-trained BERT model fine-tuned for semantic similarity tasks. By encoding input sentences into dense embeddings, SBERT can capture the semantic relationships between sentences, making it well-suited for identifying contextually similar examples even when the input phrasing differs from the training data. This capability is particularly advantageous in the biomedical domain, where synonymous terms and varied expressions are common.
3. ColBERT: ColBERT (Contextualized Late Interaction over BERT) enhances retrieval performance by focusing on contextualized token representations. It uses a late-interaction mechanism that allows for more nuanced matching of query and document tokens. We selected ColBERT for its ability to capture fine-grained semantic details, which is essential for handling complex biomedical texts with diverse and context-dependent entity mentions.
4. Dense Passage Retrieval (DPR): DPR employs a dual-encoder architecture, where separate encoders are used for queries and documents. It uses deep neural networks to learn dense embeddings, optimizing for maximum similarity between relevant query-document pairs. DPR’s strength lies in its ability to handle open-domain retrieval tasks effectively, making it a powerful choice for dynamically selecting annotated examples that are highly relevant to the input text, thus improving the contextual adaptability of our dynamic prompts.

In our experiments, we evaluated the performance of each retrieval method, assessing their impact on few-shot NER across multiple biomedical datasets.

### Retrieval pool size ablation

While our RAG-based dynamic prompting framework retrieves only a small set of in-context exemples per query, it relies on a pre-indexed pool of annotated examples. This design raises questions in scalability and cross-domain generalizability, particularly in resource-constrained scenarios. In real-world applications such as rare disease NER or cross-institutional deployments, access to large labeled datasets may be limited. To systematically assess the robustness of our framework under constrained supervision, we conducted a retrieval pool size ablation study across five biomedical NER datasets, examining the sensitivity of downstream performance to the number of annotated examples available for retrieval.

Specifically, for each benchmark dataset, we constructed separate retrieval indices using 50, 100, and 200 annotated training examples, respectively. These indices were then used to dynamically retrieve the top-*k* similar examples for few-shot inference, following the same retrieval and prompting pipeline described earlier in this section. We selected TF-IDF and SBERT as representative retrieval engines based on their strong and stable performance in earlier experiments. Due to computational constraints associated with LLaMA3-70B and its input length limits, this ablation focuses on the 5-shot and 10-shot settings. For each pool size, we performed two independent subsampling runs and report the averaged results. All ablation experiments were conducted using the LLaMA3-70B model to control for variation in backbone model capacity.

This experimental design isolates the effect of retrieval pool size on downstream performance while holding the retrieval architecture and model backbone constant. Prior work has shown that retrieval-augmented prompting can reduce labeled data requirements^36^, however, the optimal trade-off between retrieval pool sparsity and prediction quality remains underexplored in biomedical entity recognition. Moreover, prompt length constraints in LLMs^37^ further motivate evaluating whether a reduced pool of highly informative examples can approximate retrieval from full training sets. This ablation therefore provides direct insight into the annotation-efficiency of RAG-based prompting in low-resource biomedical domains.

### Experimental setup

Below, we report our experimental setup for the two prompting strategies—Static Prompting and Dynamic Prompting.

For static prompting, we evaluated three language models: GPT-3.5, GPT-4, and LLaMA 3. We used prompts containing five examples per label to provide context and guide the models’ predictions. For GPT-3.5, we used the OpenAI API version “2023-07-01-preview", and for GPT-4, we used the version “2024-02-15-preview". Both models were configured with the following settings: temperature = 0.2, top_p = 0.1, frequency_penalty = 0, presence_penalty = 0, and no stop tokens specified. The low top_p setting (0.1) was selected based on OpenAI’s recommendations and our preliminary trials, which showed that lower sampling thresholds led to more stable and deterministic outputs in biomedical NER tasks.

For LLaMA 3, we used the Meta-Llama-3-70B-Instruct model, with a temperature of 0.5 and top_p of 0.95. Lower top_p values produced repetitive or degenerate generations in early experiments, motivating the use of a higher nucleus sampling threshold. Preliminary experiments (reported in the “Results” section) revealed that GPT-3.5 consistently performed significantly worse compared to GPT-4. Hence, we excluded GPT-3.5 from further experiments in the dynamic prompting phase to limit API usage costs. To ensure robustness in the static prompting phase, the few-shot examples were randomly selected four times, and the reported results are the average of these four random selections.

In the dynamic prompting phase, we evaluated GPT-4, LLaMA 3, and the newly released open-weight GPT-OSS-120B model to explore the applicability of emerging open-source foundation models in biomedical information extraction tasks. For GPT-OSS-120B, we used a temperature of 1.0 and top_p of 1.0, consistent with the OpenAI-recommended default configuration for this model. We conducted experiments using three different in-context settings: 5-shot, 10-shot, and 20-shot, to assess the impact of increasing the number of examples on the model’s performance. The baseline prompts in this phase also used randomly selected examples, with the results averaged over four random runs.

The evaluations were conducted on five biomedical datasets: MIMIC-III (clinical notes dataset), BC5CDR (disease and chemical entity recognition), NCBI-Disease (disease annotations from PubMed abstracts), Med-Mentions (large-scale UMLS concepts dataset), and our REDDIT-IMPACTS dataset (annotated for clinical and social impacts entity extraction). Further details about these datasets are provided in the “Datasets” section. We used strict entity-level precision, recall, and micro-averaged F_1_-score (F_1_), with F_1_ as the primary evaluation metric to comprehensively assess the performance of the models’ in different datasets. Predictions were generated using a BIO tagging scheme at the token level, but evaluation was conducted at the entity level, where an entity was considered correct only if both its span and label exactly matched the ground truth. In addition, to account for the variability in performance across different experimental runs, we include 95% confidence intervals (CIs)^32^ for the reported F_1_-scores, providing a measure of the statistical robustness of the results. The confidence intervals were computed via bootstrap resampling^38^ with 1000 samples with replacement.

### Datasets

We utilized five distinct medical text datasets as benchmarks to evaluate the performance of our models and to support the development of new approaches. These datasets provide a diverse range of clinical narratives and biomedical information, allowing for a comprehensive assessment of our methods.

1. MIMIC III^39^. The MIMIC III dataset is a large, publicly available database with patient data from critical care units, including medications, lab results, clinical notes, diagnostic codes, imaging reports, and survival data. It is widely used for few-shot classification and NER tasks.
2. BC5CDR^40^. This resource extracts relationships between chemicals and diseases from annotated biomedical articles, aimed at developing systems to automatically identify these interactions for applications like drug discovery, toxicology, and understanding disease mechanisms.
3. Med-Mentions^41^. Med-Mentions is a large biomedical corpus annotated with UMLS concepts, containing PubMed articles linked to entities like diseases, chemicals, genes, and anatomical terms. It supports tasks such as information extraction, literature mining, and knowledge base construction.
4. NCBI-Disease^42^. This dataset contains PubMed abstracts annotated with disease names, linked to standardized concepts in MeSH and OMIM databases. It is used to train and evaluate models for recognizing and normalizing disease names in biomedical texts.
5. REDDIT-IMPACTS^43^. A challenging NER dataset curated from subreddits dedicated to discussions on prescription and illicit opioids, as well as medications for opioid use disorder. This dataset includes posts from 14 opioid-related subreddits, and specifically focuses on the clinical and social impacts of nonmedical substance use.

Table 4 presents relevant statistics for all publicly available datasets we used in this study, including the source and aim of each dataset, training and test set sizes, the number of entity types, and the number of entities in each dataset. None of the datasets used in this study contains Protected Health Information (PHI) or personally identifiable information. All experiments were conducted in accordance with data privacy best practices. GPT-3.5 and GPT-4 were accessed via Azure OpenAI endpoints, which provide HIPAA-compliant infrastructure when required. However, since all inputs were drawn from de-identified public benchmarks, HIPAA compliance was not a constraint in our deployment setup.

**Table 4:** Statistics of the five standardized biomedical datasets we used, including the source and aim of their tasks, training and test sizes (number of tokens), the number of entity types and the number of entities in each dataset

Datasets	Training size	Test size	Entity types	Entities
MIMIC III (information relating to patients)	36.4k	6.4k	12	8.7k
BC5CDR (extracting relationships between chemicals and diseases)	228.8k	122.2k	2	28.8k
Med-Mentions (annotated with UMLS concepts)	847.9k	593.6k	1	340.9k
NCBI Disease (PubMed abstracts annotated with disease names)	134.0k	20.5k	4	6.3k
REDDIT-IMPACTS (clinical impacts and social impacts collected from Reddit)	30.0k	6.0k	2	0.2k

## Supplementary information

Supplementary Information

## Data Availability

All datasets analyzed in this study are publicly available or accessible through established community resources. The MIMIC-III dataset is available via PhysioNet (10.1038/sdata.2016.35). The BC5CDR (10.1093/database/baw068), NCBI-Disease (10.1016/j.jbi.2013.12.006), and MedMentions datasets (https://github.com/chanzuckerberg/MedMentions) are openly released benchmark corpora. REDDIT-IMPACTS is available through the 2024 SMM4H shared task portal and can be accessed following the standard SMM4H dataset registration procedure. No new datasets were generated for this study, and none of the datasets contain Protected Health Information (PHI) or personally identifiable information.

## Code availability

The code for the proposed method, and the prompts are available at https://github.com/Yao-Ge-1218AM/RAG-based-Dynamic-Prompting/.
