# Supplementary material for: Improving few-shot named entity recognition for large language models using structured dynamic prompting with retrieval augmented generation
Source: NPJ Artif Intell. 2026 Mar 31;2(1):39. doi: 10.1038/s44387-025-00062-2 (PMC13038409; doi:10.1038/s44387-025-00062-2)
Supplement: Supplementary file 1 — Supplementary Information [file 44387_2025_62_MOESM1_ESM.pdf]

A Averaged Performance of the Baseline Dynamic Prompt Model

	GPT-4			Llama 3			GPT-OSS		
	**Precision**	**Recall**	**F_1-scores**	**Precision**	**Recall**	**F_1-scores**	**Precision**	**Recall**	**F_1-scores**
**5-shot**	16.40	50.00	24.70	12.56	56.32	20.55	11.61	30.19	16.77
	17.67	50.62	26.20	13.68	55.81	21.97	9.41	25.47	14.06
	17.48	53.09	26.30	12.14	59.52	20.16	10.08	53.42	16.96
	23.91	54.32	33.20	14.25	59.79	23.02	10.53	27.18	15.18
**AVG**	18.865	52.0075	27.60	13.1575	57.86	21.425	10.4075	34.065	15.7425
**10-shot**	25.41	58.75	35.47	24.51	59.52	34.72	11.21	39.87	17.50
	21.67	54.32	30.99	19.63	62.35	29.86	9.90	33.56	15.29
	21.43	59.26	31.48	23.96	57.44	33.81	11.62	53.14	19.07
	20.47	54.32	29.73	21.38	60.43	31.59	10.12	41.34	16.26
**AVG**	22.245	56.6625	31.9175	22.37	59.935	32.495	10.7125	41.9775	17.03
**20-shot**	28.74	58.63	38.57	27.22	57.65	36.98	15.84	48.08	23.83
	27.03	57.54	36.78	23.24	52.44	32.21	13.28	47.96	20.80
	26.52	59.79	36.74	22.12	52.08	31.06	13.27	36.83	19.51
	28.65	59.02	38.57	25.49	53.06	34.44	13.47	42.62	20.47
**AVG**	27.735	58.745	37.665	24.5175	53.8075	33.6725	13.965	43.8725	21.1525

Table S1. Averaged performance of the baseline dynamic prompt model on the REDDIT-IMPACTS dataset across different shot settings.

	GPT-4			Llama 3			GPT-OSS		
	Precision	Recall	F_1-scores	Precision	Recall	F_1-scores	Precision	Recall	F_1-scores
5-shot	69.97	91.43	79.27	68.09	84.35	75.35	70.82	92.43	80.19
	68.32	88.3	77.29	69.70	76.67	73.02	75.18	81.50	78.21
	64.58	90.15	75.25	71.00	79.15	74.86	76.05	76.99	76.52
	71.59	91.39	80.29	67.10	73.25	70.04	75.38	83.63	79.29
AVG	68.615	90.3175	78.025	68.9725	78.355	73.3175	74.3575	83.6375	78.5525
10-shot	75.11	90.84	82.23	72.38	79.18	75.63	78.33	84.42	81.26
	74.41	90.32	81.60	73.31	74.24	73.77	77.79	87.61	82.41
	74.13	85.71	79.50	71.15	77.05	73.98	76.63	88.46	82.12
	77.65	86.35	81.73	73.40	81.17	77.2	78.04	84.34	81.07
AVG	75.325	88.305	81.265	72.56	77.91	75.145	77.6975	86.2075	81.715
20-shot	75.36	88.33	81.33	75.12	74.84	74.98	77.66	87.17	82.14
	73.13	91.74	81.38	72.03	71.88	71.96	78.13	83.00	80.49
	71.84	91.1	80.33	76.88	77.20	77.04	78.22	89.41	83.44
	77.94	85.54	81.57	77.64	78.40	78.02	78.81	86.68	82.56
AVG	74.5675	89.1775	81.1525	75.4175	75.58	75.5	78.205	86.565	82.1575

Table S2. Averaged performance of the baseline dynamic prompt model on the BC5CDR dataset across different shot settings.

	GPT-4			Llama 3			GPT-OSS		
	Precision	Recall	F_1-scores	Precision	Recall	F_1-scores	Precision	Recall	F_1-scores
5-shot	62.38	63.78	63.07	57.69	68.39	62.58	50.60	52.29	51.43
	61.88	62.19	62.03	61.83	61.33	61.58	50.92	51.24	51.08
	65.26	68.14	66.67	58.24	68.06	62.77	49.55	46.86	48.07
	62.71	62.36	62.54	59.44	71.29	64.82	51.68	48.31	49.94
AVG	63.0575	64.1175	63.5775	59.3	67.2675	62.9375	50.6875	49.675	50.13
10-shot	66.38	74.24	70.09	59.13	71.63	64.78	56.55	58.81	57.66
	68.37	74.86	71.47	62.68	63.8	63.23	54.75	56.54	55.63
	66.72	75.33	70.77	61.71	67.58	64.51	55.17	57.98	56.54
	66.46	73.34	69.73	62.24	62.84	62.54	52.34	54.57	53.43
AVG	66.9825	74.4425	70.515	61.44	66.4625	63.765	54.7025	56.975	55.815
20-shot	70.41	69.84	70.12	63.89	62.04	62.95	56.18	54.95	55.56
	70.11	71.24	70.67	63.96	62.83	63.39	56.31	60.87	58.50
	71.45	73.39	72.41	59.22	61.71	60.44	56.39	59.62	57.96
	70.64	70.80	70.72	60.96	61.88	61.42	56.56	58.08	57.31
AVG	70.6525	71.3175	70.98	62.0075	62.115	62.05	56.36	58.38	57.3325

Table S3. Averaged performance of the baseline dynamic prompt model on the MIMIC III dataset across different shot settings.

	GPT-4			Llama 3			GPT-OSS		
	**Precision**	**Recall**	**F_1-scores**	**Precision**	**Recall**	**F_1-scores**	**Precision**	**Recall**	**F_1-scores**
**5-shot**	46.17	50.52	48.25	33.88	40.67	36.97	36.70	32.04	34.21
	45.69	49.07	47.32	32.26	39.14	35.37	35.51	37.29	36.38
	40.24	43.65	41.88	36.38	28.49	31.95	35.38	39.87	37.49
	47.96	52.83	50.28	40.73	30.53	34.9	38.53	36.49	37.48
**AVG**	45.015	49.0175	46.9325	35.8125	34.7075	34.7975	36.53	36.4225	36.39
**10-shot**	52.98	52.67	52.82	42.79	31.08	35.66	37.26	36.16	36.70
	53.71	53.36	53.54	40.18	28.73	36.27	40.19	31.84	35.53
	52.99	50.35	51.64	35.75	32.05	33.76	34.91	34.85	34.88
	53.85	52.3	53.06	39.95	34.11	36.71	36.54	34.31	35.39
**AVG**	53.3825	52.17	52.765	39.6675	31.4925	35.6	37.225	34.29	35.625
**20-shot**	54.57	54.73	54.65	40.59	42.07	41.32	37.92	39.02	38.46
	44.86	45.43	45.14	40.87	42.8	41.81	38.23	41.68	39.88
	51.6	51.75	51.68	41.48	43.02	42.24	37.83	40.07	38.92
	55.7	57.24	56.46	39.88	42.41	41.1	36.29	40.52	38.29
**AVG**	51.6825	52.2875	51.9825	40.705	42.575	41.6175	37.5675	40.3225	38.8875

Table S4. Averaged performance of the baseline dynamic prompt model on the NCBI dataset across different shot settings.

	GPT-4			Llama 3			GPT-OSS		
	**Precision**	**Recall**	**F_1-scores**	**Precision**	**Recall**	**F_1-scores**	**Precision**	**Recall**	**F_1-scores**
**5-shot**	27.24	62.73	37.99	24.94	73.03	37.18	15.05	57.28	23.84
	26.71	58.58	36.69	25.95	61.96	36.58	13.92	59.63	22.57
	29.23	60.47	39.41	26.11	73.57	38.54	13.47	55.98	21.71
	25.86	58.41	35.85	26.55	59.62	36.74	15.73	53.63	24.33
**AVG**	27.26	60.0475	37.485	25.8875	67.045	37.26	14.5425	56.63	23.1125
**10-shot**	26.92	58.26	36.82	25.67	70.61	37.65	17.13	57.87	26.44
	26.1	59.15	36.22	25.1	59.19	35.15	15.61	56.55	24.47
	24.41	58.56	34.46	25.76	70.06	37.67	17.61	59.27	27.15
	29.19	60.82	39.45	25.73	57.46	35.54	17.09	56.33	26.22
**AVG**	26.655	59.1975	36.7375	25.565	64.33	36.5025	16.86	57.505	26.07
**20-shot**	27.86	59.12	37.87	26.18	63.59	37.09	19.22	60.56	29.18
	25.02	60.64	35.42	26.63	61.14	37.1	19.85	61.64	30.03
	29.33	61.71	39.76	25.93	63.57	36.84	17.99	57.58	27.41
	30.18	61.66	40.52	27.54	70.85	39.66	18.28	59.83	28.00
**AVG**	28.0975	60.7825	38.3925	26.57	64.7875	37.6725	18.835	59.9025	28.655

Table S5. Averaged performance of the baseline dynamic prompt model on the Med-Mentions dataset across different shot settings.

B Results of Precision, Recall and 95% CIs for F_1-scores

	Reddit_Impacts			*BC5CDR*			*MIMIC III*			*NCBI*			*Med-Mentions*		
	**P**	**R**	**F_1**	**P**	**R**	**F_1**	**P**	**R**	**F_1**	**P**	**R**	**F_1**	**P**	**R**	**F_1**
**GPT-3.5**															
**Basic Prompt (BP)**	10.37	43.26	16.73	55.64	76.88	64.56	55.31	54.11	54.70	18.28	51.33	26.96	8.55	10.12	9.27
**BP + Description of datasets**	13.25	52.38	21.15	59.25	81.47	68.61	59.54	54.18	56.73	26.24	50.25	34.48	16.52	10.33	12.71
**BP + High-frequency instances**	13.40	50.13	21.15	59.08	82.96	69.01	59.93	55.66	57.72	26.54	55.68	35.95	20.16	15.03	17.22
**BP + UMLS knowledge**	10.17	42.86	**16.44**	54.24	80.57	64.83	47.16	54.50	**50.57**	23.93	43.02	30.75	10.26	11.58	10.88
**BP + Error analysis**	12.02	48.21	19.24	57.36	82.49	67.67	64.63	55.16	59.52	25.23	48.31	33.15	18.74	13.24	15.52
**BP + 5-shot learning with sentences**	12.33	44.42	19.30	59.30	82.04	68.84	53.09	53.37	57.03	37.09	43.78	40.16	17.28	25.54	20.61
**BP + 5-shot learning with tokens**	**13.63**	53.11	**21.69**	**62.27**	82.02	**70.79**	**67.50**	55.97	**61.21**	**40.15**	46.32	**43.01**	**20.54**	30.57	**24.57**
**BP + All above**	**15.36**	**53.92**	**23.91**	**63.64**	**84.86**	**72.73**	**67.77**	**57.10**	**61.99**	**42.73**	**48.07**	**45.24**	**22.15**	**55.32**	**31.63**
**GPT-4**															
**Basic Prompt (BP)**	12.75	48.15	20.16	59.56	83.22	69.43	57.57	55.72	56.63	25.13	50.48	33.56	18.27	11.12	13.83
**BP + Description of datasets**	15.12	52.94	23.52	60.66	84.58	70.65	63.35	56.42	59.68	26.43	55.22	35.75	21.23	11.96	15.30
**BP + High-frequency instances**	15.98	53.75	24.64	63.89	84.06	72.60	**64.61**	56.14	60.08	35.02	41.44	37.96	21.72	17.69	19.50
**BP + UMLS knowledge**	12.85	50.14	20.46	59.48	84.63	69.86	55.37	54.90	**55.13**	22.80	47.92	**30.90**	18.72	11.83	14.50
**BP + Error analysis**	14.87	52.04	23.13	67.92	82.75	74.61	63.93	56.72	60.11	34.86	41.38	37.84	20.28	16.59	18.25
**BP + 5-shot learning with sentences**	14.71	51.48	22.88	65.04	83.18	73.00	58.49	55.03	58.25	36.96	45.67	40.86	27.42	30.33	28.80
**BP + 5-shot learning with tokens**	**17.23**	52.57	**25.95**	**68.10**	87.66	**76.65**	63.40	62.49	**62.94**	**40.72**	48.42	**44.24**	**27.71**	41.41	**33.20**
**BP + All above**	**18.87**	**52.01**	**27.60**	**68.62**	**90.32**	**78.03**	63.06	**64.12**	**63.58**	**45.02**	49.02	**46.93**	27.26	**60.06**	**37.49**
**Llama3-70B**															
**Basic Prompt (BP)**	9.93	36.42	15.61	52.52	76.04	62.13	46.57	55.64	50.70	15.63	24.37	19.15	19.59	23.17	21.23
**BP + Description of datasets**	13.27	35.26	19.28	58.53	80.22	67.68	56.64	55.81	56.22	17.30	36.43	21.44	23.59	19.87	21.57
**BP + High-frequency instances**	**14.52**	34.53	**20.44**	60.85	78.05	68.39	55.65	56.47	56.06	21.11	42.97	26.62	**23.99**	31.20	27.12
**BP + UMLS knowledge**	7.87	35.88	**12.91**	57.11	74.63	64.71	46.78	51.63	**48.92**	14.95	34.72	20.91	22.09	25.51	23.68
**BP + Error analysis**	12.46	38.86	18.87	58.96	80.52	68.07	63.18	55.20	58.92	16.84	44.65	24.46	22.64	29.92	25.78
**BP + 5-shot learning with sentences**	11.35	39.71	17.65	65.16	77.28	70.70	62.90	51.61	56.85	21.97	49.94	30.52	23.87	64.66	34.87
**BP + 5-shot learning with tokens**	13.33	40.32	20.04	**66.03**	78.57	**71.76**	**63.89**	60.19	**61.98**	**34.49**	32.41	**33.42**	23.72	68.45	**35.23**
**BP + All above**	13.16	**57.86**	**21.43**	**68.97**	78.36	**73.32**	59.30	**67.27**	**62.94**	**35.81**	**34.71**	**34.80**	**25.89**	**67.05**	**37.26**

Table S6. Performance comparison of various prompting strategies across different datasets in terms of F_1-score (F_1), Precision (P), and Recall (R). The row "BP + All components" represents the combination of all strategies, with the best performance across datasets highlighted in bold. The **red bold** indicates the best F_1 score achieved by an individual component, while **black bold** font highlights the highest Precision, and underlined text denotes the best Recall for a single component. Additionally, **green bold** is used to mark F_1 scores that are lower than the baseline performance (BP).

	Reddit_Impacts	*BC5CDR*	*MIMIC III*	*NCBI*	*Med-Mentions*
**GPT-3.5**					
**Basic Prompt (BP)**	16.73 [11.53, 22.83]	64.56 [61.55, 67.73]	54.70 [49.60, 58.73]	26.96 [24.43, 30.98]	9.27 [7.81, 12.22]
**BP + Description of datasets**	21.15 [14.88, 26.64]	68.61 [66.74, 70.72]	56.73 [52.58, 61.22]	34.48 [31.08, 39.25]	12.71 [10.93, 15.65]
**BP + High-frequency instances**	21.15 [15.75, 27.40]	69.01 [66.24, 70.98]	57.72 [52.75, 62.26]	35.95 [33.36, 38.44]	17.22 [14.47, 19.80]
**BP + UMLS knowledge**	16.44 [8.43, 23.07]	64.83 [61.83, 66.41]	50.57 [46.17, 55.04]	30.75 [27.73, 33.26]	10.88 [8.81, 12.29]
**BP + Error analysis**	19.24 [12.91, 26.17]	67.67 [65.53, 70.32]	59.52 [54.96, 64.47]	33.15 [31.24, 38.87]	15.52 [13.14, 17.20]
**BP + 5-shot learning with sentences**	19.30 [12.26, 25.78]	68.84 [67.25, 70.49]	57.03 [53.06, 62.85]	40.16 [38.78, 46.45]	20.61 [17.58, 22.29]
**BP + 5-shot learning with tokens**	**21.69 [15.92, 28.89]**	**70.79 [68.87, 73.15]**	**61.21 [56.81, 66.05]**	**43.01 [41.43, 48.21]**	**24.57 [22.88, 26.64]**
**BP + All above**	**23.91 [15.87, 30.97]**	**72.73 [70.32, 74.86]**	**61.99 [57.24, 66.38]**	**45.24 [42.64, 50.58]**	**31.63 [29.36, 34.74]**
**GPT-4**					
**Basic Prompt (BP)**	20.16 [13.29, 26.54]	69.43 [66.28, 72.44]	56.63 [51.27, 60.83]	33.56 [31.59, 37.25]	13.83 [11.85, 15.09]
**BP + Description of datasets**	23.52 [16.46, 30.84]	70.65 [67.47, 72.72]	59.68 [55.18, 64.09]	35.75 [33.54, 40.58]	15.30 [13.61, 17.15]
**BP + High-frequency instances**	24.64 [17.72, 31.11]	72.60 [71.17, 74.28]	60.08 [56.33, 65.37]	37.96 [36.95, 41.73]	19.50 [17.11, 22.97]
**BP + UMLS knowledge**	20.46 [13.84, 27.07]	69.86 [66.05, 72.62]	55.13 [50.20, 60.29]	30.90 [28.68, 34.30]	14.50 [12.57, 16.46]
**BP + Error analysis**	23.13 [16.65, 30.69]	74.61 [71.44, 77.29]	60.11 [55.44, 64.72]	37.84 [34.13, 42.71]	18.25 [15.06, 20.43]
**BP + 5-shot learning with sentences**	22.88 [16.23, 30.59]	73.00 [71.26, 76.22]	58.25 [53.28, 63.95]	40.86 [39.37, 45.36]	28.80 [26.71, 30.20]
**BP + 5-shot learning with tokens**	**25.95 [18.50, 32.07]**	**76.65 [74.15, 77.92]**	**62.94 [57.56, 66.87]**	**44.24 [42.93, 48.28]**	**33.20 [31.64, 35.70]**
**BP + All above**	**27.60 [19.43, 33.80]**	**78.03 [75.51, 80.02]**	**63.58 [58.73, 67.18]**	**46.93 [44.85, 51.58]**	**37.95 [35.88, 39.90]**
**Llama3**					
**Basic Prompt (BP)**	15.61 [8.20, 22.12]	62.13 [59.24, 63.58]	50.70 [45.93, 54.19]	19.15 [15.21, 21.38]	21.23 [19.24, 23.42]
**BP + Description of datasets**	19.28 [11.71, 25.96]	67.68 [64.86, 69.10]	56.22 [52.77, 60.25]	21.44 [20.80, 24.65]	21.57 [19.30, 24.76]
**BP + High-frequency instances**	**20.44 [13.79, 27.51]**	68.39 [66.48, 70.35]	56.06 [52.62, 61.42]	26.62 [22.16, 28.31]	27.12 [26.37, 29.35]
**BP + UMLS knowledge**	12.91 [7.40, 18.71]	64.71 [61.44, 67.01]	48.92 [44.75, 53.37]	20.91 [17.07, 22.61]	23.68 [20.59, 25.17]
**BP + Error analysis**	18.87 [13.34, 25.13]	68.07 [65.41, 70.58]	58.92 [53.90, 63.84]	24.46 [20.97, 25.20]	25.78 [23.48, 27.56]
**BP + 5-shot learning with sentences**	17.65 [13.62, 24.69]	70.70 [69.36, 72.83]	56.85 [52.32, 61.33]	30.52 [26.50, 33.96]	34.87 [32.18, 37.25]
**BP + 5-shot learning with tokens**	20.04 [14.81, 27.29]	**71.76 [69.58, 73.51]**	**61.98 [56.59, 65.18]**	**33.42 [28.72, 35.12]**	**35.23 [33.17, 37.08]**
**BP + All above**	**21.43 [14.24, 28.80]**	**73.32 [72.27, 74.26]**	**62.94 [57.07, 65.79]**	**34.80 [28.57, 35.44]**	**37.26 [35.45, 39.08]**

Table S7. Evaluation of static prompting strategies using GPT-3.5, GPT-4 and Llama 3 across five biomedical datasets. The table presents F_1-score with 95% confidence intervals reported for the reported F_1-score to indicate the statistical reliability of the results.

		Reddit_Impacts			*BC5CDR*			*MIMIC III*			*NCBI*			*Med-Mentions*		
		**P**	**R**	**F_1**	**P**	**R**	**F_1**	**P**	**R**	**F_1**	**P**	**R**	**F_1**	**P**	**R**	**F_1**
**GPT-4**																
5-shot	**Base**	18.87	52.01	27.60	68.62	90.32	78.03	63.06	64.12	63.58	45.02	49.02	46.93	27.26	60.06	37.49
	**TF-IDF**	19.71	51.25	28.47	**82.31**	89.76	**85.88**	**74.43**	78.14	**76.24**	**56.86**	63.68	**60.08**	27.22	62.68	37.96
	**SBERT**	**24.31**	55.00	**33.72**	76.63	91.41	83.37	72.63	74.27	73.44	55.05	60.30	57.56	28.05	64.65	39.12
	**ColBERT**	22.66	56.79	32.39	78.64	81.03	79.82	74.14	77.02	75.56	50.43	54.48	52.38	**28.14**	68.69	**39.93**
	**DPR**	22.60	58.79	32.64	79.39	88.24	83.58	69.77	70.00	69.89	46.67	52.39	49.37	27.90	65.49	39.13
10-shot	**Base**	22.25	56.66	31.92	75.33	88.31	81.27	66.38	74.24	70.09	53.23	52.13	52.67	26.67	59.20	36.74
	**TF-IDF**	21.53	56.25	31.14	83.81	89.67	**86.64**	73.85	77.29	75.53	**58.81**	65.66	**62.05**	28.14	71.42	40.37
	**SBERT**	**25.41**	58.75	**35.47**	83.94	87.99	85.92	72.73	75.08	73.89	58.79	63.02	60.83	**28.32**	70.26	40.37
	**ColBERT**	23.86	58.02	33.81	83.49	88.05	85.71	**74.69**	78.06	**76.34**	55.12	59.56	57.25	28.15	71.99	**40.48**
	**DPR**	22.96	56.25	32.61	**85.16**	84.42	84.79	71.84	72.42	72.13	56.82	60.72	58.70	28.25	70.04	40.25
20-shot	**Base**	27.74	58.75	37.67	74.57	89.18	81.15	70.65	71.32	70.98	51.68	52.29	51.98	28.10	60.78	38.39
	**TF-IDF**	27.72	62.20	38.35	85.41	88.98	87.16	**75.81**	79.61	**77.66**	**61.80**	67.13	**64.36**	**28.20**	77.30	**41.32**
	**SBERT**	28.44	59.50	38.22	85.37	89.57	**87.42**	73.79	76.54	75.14	60.89	63.59	62.21	26.81	74.09	39.37
	**ColBERT**	**31.19**	66.67	**42.49**	82.09	83.94	83.00	75.27	78.19	76.70	56.13	59.35	57.69	27.70	75.47	40.53
	**DPR**	28.55	60.75	38.84	**85.81**	85.40	85.60	71.82	72.74	72.28	59.00	61.74	60.34	27.16	69.37	39.23

Table S8. Evaluation of dynamic prompting strategies (5-shot, 10-shot, and 20-shot) using GPT-4 across five biomedical datasets. The table presents F_1-score, precision, and recall for each retrieval method: Base Prompt, TF-IDF, SBERT, ColBERT, and DPR. The row "Base" represents using static prompts we proposed in the former section.

		Reddit_Impacts			*BC5CDR*			*MIMIC III*			*NCBI*			*Med-Mentions*		
		**P**	**R**	**F_1**	**P**	**R**	**F_1**	**P**	**R**	**F_1**	**P**	**R**	**F_1**	**P**	**R**	**F_1**
**Llama3-70B**																
5-shot	**Base**	13.16	57.86	21.43	68.97	78.36	73.32	59.30	67.27	62.94	35.81	34.71	34.80	25.89	67.05	37.26
	**TF-IDF**	18.89	58.62	28.57	**78.49**	81.78	80.11	66.48	74.84	70.41	48.93	50.70	49.80	26.46	72.06	38.68
	**SBERT**	**23.20**	66.67	**34.42**	77.26	83.79	**80.39**	64.04	72.21	67.88	**50.66**	49.59	**50.12**	26.15	68.92	37.91
	**ColBERT**	22.05	65.12	32.94	71.21	72.33	71.76	**68.37**	75.32	**71.68**	44.93	46.08	45.50	**26.68**	72.38	**38.99**
	**DPR**	19.20	59.26	29.00	74.47	76.91	75.67	65.74	72.54	68.97	41.06	48.66	44.54	26.51	71.38	38.66
10-shot	**Base**	22.37	59.94	32.50	72.56	77.91	75.15	59.13	71.63	63.77	39.67	31.49	35.60	25.57	64.33	36.50
	**TF-IDF**	23.53	62.65	34.21	80.82	80.32	80.57	55.79	55.34	55.56	49.59	49.41	49.50	24.03	68.00	35.51
	**SBERT**	22.27	59.76	32.45	77.72	84.94	81.17	67.67	76.09	71.63	**52.84**	49.94	**51.35**	**27.61**	66.88	**39.08**
	**ColBERT**	22.58	60.50	32.89	78.40	82.37	80.34	**69.65**	76.37	**72.85**	38.72	38.81	38.77	26.49	67.58	38.06
	**DPR**	**24.37**	57.83	**34.29**	**85.16**	84.42	**84.79**	65.85	73.68	69.54	47.60	45.04	46.28	25.80	70.97	37.85
20-shot	**Base**	24.52	53.81	33.67	**75.42**	75.58	75.50	62.01	62.12	62.05	40.71	42.58	41.62	26.57	64.79	37.67
	**TF-IDF**	27.62	66.95	39.11	74.64	82.47	**78.36**	55.95	58.51	57.66	45.39	49.83	47.50	**27.80**	64.39	**38.83**
	**SBERT**	**29.93**	68.06	**41.43**	75.04	80.75	76.85	**65.90**	64.77	65.35	42.09	46.40	44.14	25.48	61.36	36.01
	**ColBERT**	23.57	65.48	34.66	73.74	70.70	72.19	58.25	57.03	57.63	**47.08**	49.88	**48.44**	25.41	66.98	36.85
	**DPR**	26.15	65.04	37.30	72.58	77.15	74.80	62.72	69.19	**65.80**	37.18	44.13	40.36	26.10	62.88	36.89

Table S9. Evaluation of dynamic prompting strategies (5-shot, 10-shot, and 20-shot) using Llama 3 across five biomedical datasets. The table presents F_1-score, precision, and recall for each retrieval method: Base Prompt, TF-IDF, SBERT, ColBERT, and DPR. The row "Base" represents using static prompts we proposed in the former section.

		Reddit_Impacts			*BC5CDR*			*MIMIC III*			*NCBI*			*Med-Mentions*		
		**P**	**R**	**F_1**	**P**	**R**	**F_1**	**P**	**R**	**F_1**	**P**	**R**	**F_1**	**P**	**R**	**F_1**
**GPT-OSS**																
5-shot	**Base**	10.26	33.79	15.74	75.41	81.96	78.55	54.03	46.75	50.13	32.83	40.82	36.39	22.76	23.47	23.11
	**TF-IDF**	13.17	46.25	20.50	82.61	89.68	**86.00**	**68.15**	71.89	**69.97**	50.29	60.41	**54.89**	**25.04**	45.45	**32.29**
	**SBERT**	**14.75**	39.51	21.48	81.64	87.11	84.29	66.03	67.31	66.67	49.18	56.80	52.72	24.63	40.62	30.67
	**ColBERT**	14.44	48.19	22.22	**82.84**	88.30	85.48	67.49	70.55	68.99	47.04	50.93	48.91	24.30	38.47	29.79
	**DPR**	14.55	50.00	**22.54**	80.96	86.61	83.69	67.32	70.53	68.89	**52.22**	56.55	54.30	24.76	33.15	28.35
10-shot	**Base**	11.48	32.79	17.03	78.49	85.23	81.72	62.19	50.63	55.82	32.44	39.45	35.60	23.65	29.04	26.07
	**TF-IDF**	16.35	52.46	24.93	**84.41**	91.54	**87.83**	69.72	73.25	71.44	**50.65**	60.22	**55.02**	**27.42**	56.73	**36.97**
	**SBERT**	**16.67**	53.69	25.43	82.43	83.19	85.21	66.64	70.02	68.29	49.24	57.15	52.90	24.32	45.42	31.68
	**ColBERT**	16.42	53.01	25.07	47.19	48.82	47.99	**70.46**	74.41	**72.38**	48.73	51.63	50.14	24.72	46.49	32.27
	**DPR**	16.61	58.54	**26.88**	81.69	87.70	84.58	68.81	72.19	70.46	49.26	56.83	52.77	23.65	36.57	28.72
20-shot	**Base**	13.63	47.18	21.15	79.38	85.14	82.16	63.33	49.90	57.33	35.13	43.56	38.89	24.22	35.09	28.66
	**TF-IDF**	18.39	53.41	27.36	82.94	90.36	**86.49**	**71.21**	76.25	**73.69**	**53.71**	63.58	**58.23**	**26.32**	55.08	**35.62**
	**SBERT**	19.74	61.19	29.85	**83.49**	89.57	86.42	68.54	72.26	70.35	52.96	56.73	54.78	26.10	52.07	34.77
	**ColBERT**	**20.07**	62.64	**30.40**	48.07	50.20	49.11	71.15	76.03	73.51	52.19	55.19	53.65	24.84	52.13	33.65
	**DPR**	19.45	63.42	29.77	80.54	88.39	84.28	67.87	70.21	69.02	53.64	61.77	57.42	25.54	42.81	31.99

Table S10. Evaluation of dynamic prompting strategies (5-shot, 10-shot, and 20-shot) using GPT-OSS across five biomedical datasets. The table presents F_1-score, precision, and recall for each retrieval method: Base Prompt, TF-IDF, SBERT, ColBERT, and DPR. The row "Base" represents using static prompts we proposed in the former section.

		Reddit_Impacts	*BC5CDR*	*MIMIC III*	*NCBI*	*Med-Mentions*
**GPT-4**						
5-shot	**Base**	27.60 [19.43, 33.80]	78.03 [75.51, 80.02]	63.58 [58.73, 67.18]	46.93 [44.85, 51.58]	37.95 [35.88, 39.90]
	**TF-IDF**	28.47 [21.78, 35.47]	**85.88 [84.53, 86.42]**	**76.24 [72.98, 79.63]**	**60.08 [56.70, 63.32]**	37.96 [35.90, 39.84]
	**SBERT**	**33.72 [26.28, 42.20]**	83.37 [82.51, 84.22]	73.44 [69.91, 76.81]	57.56 [54.05, 60.73]	39.12 [36.84, 41.34]
	**ColBERT**	32.39 [25.10, 39.85]	79.82 [78.24, 80.98]	75.56 [72.06, 78.94]	52.38 [49.06, 55.55]	**39.93 [37.93, 41.73]**
	**DPR**	32.64 [25.42, 40.17]	83.58 [82.30, 84.88]	69.89 [65.75, 73.63]	49.37 [45.37, 52.94]	39.13 [34.44, 41.35]
10-shot	**Base**	31.92 [23.77, 38.44]	81.27 [80.81, 82.37]	70.52 [66.10, 73.81]	52.67 [49.36, 56.76]	36.74 [32.29, 38.83]
	**TF-IDF**	31.14 [24.33, 38.13]	**86.64 [85.15, 88.09]**	75.53 [72.18, 79.10]	**62.05 [58.79, 65.11]**	40.37 [38.23, 42.43]
	**SBERT**	**35.47 [27.17, 43.21]**	85.92 [83.09, 87.27]	73.89 [70.22, 77.80]	60.83 [57.47, 64.03]	40.37 [38.23, 42.39]
	**ColBERT**	33.81 [26.24, 41.55]	85.71 [84.42, 86.07]	**76.34 [73.01, 79.68]**	57.25 [53.75, 60.72]	**40.48 [38.13, 42.54]**
	**DPR**	32.61 [24.50, 40.33]	84.79 [82.96, 86.78]	72.13 [68.06, 75.85]	58.70 [54.99, 61.91]	40.25 [30.83, 50.75]
20-shot	**Base**	37.67 [30.04, 43.44]	81.15 [80.40, 82.24]	70.98 [65.77, 73.82]	51.98 [50.33, 58.84]	38.39 [35.26, 40.29]
	**TF-IDF**	38.35 [30.77, 46.28]	87.16 [85.77, 88.62]	**77.66 [71.91, 78.88]**	**64.36 [61.18, 67.87]**	**41.32 [39.21, 43.26]**
	**SBERT**	38.22 [28.57, 44.90]	**87.42 [85.26, 89.12]**	75.14 [71.77, 78.75]	62.21 [59.01, 65.18]	39.37 [35.05, 40.39]
	**ColBERT**	**42.49 [32.52, 48.33]**	83.00 [81.39, 84.40]	76.70 [73.11, 79.89]	57.69 [54.22, 61.18]	40.53 [37.61, 43.26]
	**DPR**	38.84 [29.01, 44.44]	85.60 [84.28, 86.93]	72.28 [68.56, 75.95]	60.34 [56.54, 63.72]	39.23 [34.22, 41.56]

Table S11. Evaluation of dynamic prompting strategies (5-shot, 10-shot, and 20-shot) using GPT-4 across five biomedical datasets. The table presents F_1-score for each retrieval method: Base Prompt, TF-IDF, SBERT, ColBERT, and DPR, with 95% confidence intervals reported for each metric to indicate the statistical reliability of the results.

		Reddit_Impacts	*BC5CDR*	*MIMIC III*	*NCBI*	*Med-Mentions*
**Llama 3**						
5-shot	**Base**	21.43 [14.24, 28.80]	73.32 [72.27, 74.26]	62.94 [57.07, 65.79]	34.80 [28.57, 35.44]	37.26 [35.45, 39.08]
	**TF-IDF**	28.57 [21.74, 36.06]	80.11 [79.25, 81.00]	70.41 [66.87, 73.76]	49.80 [46.38, 53.03]	38.68 [35.67, 40.81]
	**SBERT**	**34.42 [26.28, 41.52]**	**80.39 [79.50, 81.33]**	67.88 [64.09, 71.69]	**50.12 [46.89, 53.66]**	37.91 [36.02, 39.81]
	**ColBERT**	32.94 [25.00, 39.84]	71.76 [70.75, 72.69]	**71.68 [68.08, 75.21]**	45.50 [41.95, 49.49]	**38.99 [36.15, 41.34]**
	**DPR**	29.00 [22.86, 36.36]	75.67 [74.67, 76.70]	68.97 [65.05, 72.70]	44.54 [41.24, 48.25]	38.66 [36.78, 40.50]
10-shot	**Base**	32.50 [26.94, 42.26]	75.15 [74.65, 76.67]	63.77 [58.59, 67.75]	35.60 [32.17, 39.12]	36.50 [35.73, 39.57]
	**TF-IDF**	34.21 [27.24, 42.03]	**80.57 [79.65, 81.47]**	55.56 [53.11, 60.44]	49.50 [46.05, 52.92]	35.51 [34.75, 37.45]
	**SBERT**	32.45 [25.33, 39.63]	81.17 [80.26, 82.03]	71.63 [67.75, 75.15]	**51.35 [47.49, 55.16]**	**39.08 [36.39, 41.38]**
	**ColBERT**	32.89 [20.35, 35.05]	80.34 [79.53, 81.24]	**72.85 [69.46, 76.49]**	38.77 [34.91, 42.29]	38.06 [35.52, 40.71]
	**DPR**	**34.29 [26.11, 41.98]**	74.72 [73.77, 75.73]	69.54 [65.61, 73.17]	46.28 [42.77, 49.65]	37.85 [36.06, 39.69]
20-shot	**Base**	33.67 [24.09, 40.88]	75.50 [73.57, 76.36]	62.05 [58.23, 67.15]	41.62 [38.83, 45.71]	37.67 [35.22, 40.57]
	**TF-IDF**	39.11 [31.34, 47.70]	**78.36 [77.42, 79.30]**	57.66 [51.19, 59.80]	47.50 [43.87, 50.84]	**38.83 [37.54, 39.11]**
	**SBERT**	**41.43 [31.58, 48.98]**	76.85 [74.86, 78.96]	65.35 [60.44, 70.40]	44.14 [40.57, 47.86]	36.01 [34.16, 37.75]
	**ColBERT**	34.66 [24.07, 36.31]	72.19 [71.17, 73.20]	57.63 [53.19, 61.93]	**48.44 [45.07, 51.78]**	36.85 [34.10, 39.29]
	**DPR**	37.30 [27.13, 44.76]	74.80 [72.49, 76.36]	**65.80 [61.82, 69.69]**	40.36 [36.96, 43.96]	36.89 [34.46, 38.84]

Table S12. Evaluation of dynamic prompting strategies (5-shot, 10-shot, and 20-shot) using Llama 3 across five biomedical datasets. The table presents F_1-score for each retrieval method: Base Prompt, TF-IDF, SBERT, ColBERT, and DPR, with 95% confidence intervals reported for each metric to indicate the statistical reliability of the results.

		Reddit_Impacts	*BC5CDR*	*MIMIC III*	*NCBI*	*Med-Mentions*
**GPT-OSS**						
5-shot	**Base**	15.74 [11.84, 21.61]	78.55 [75.26, 80.25]	50.13 [45.45, 54.77]	36.39 [31.89, 40.81]	23.11 [21.70, 25.09]
	**TF-IDF**	20.50 [15.17, 26.38]	**86.00 [83.95, 87.96]**	**69.97 [65.87, 73.82]**	**54.89 [51.31, 58.41]**	**32.29 [30.34, 34.92]**
	**SBERT**	21.48 [14.94, 28.83]	84.29 [81.92, 86.70]	66.67 [62.67, 70.61]	52.72 [48.74, 56.68]	30.67 [28.87, 32.47]
	**ColBERT**	22.22 [16.15, 28.84]	85.48 [83.39, 87.71]	68.99 [64.91, 72.69]	48.91 [46.76, 52.52]	29.79 [27.93, 31.45]
	**DPR**	**22.54 [16.71, 29.52]**	83.69 [81.31, 86.05]	68.89 [65.00, 72.78]	54.30 [51.21, 58.16]	28.35 [26.35, 30.09]
10-shot	**Base**	17.03 [12.26, 23.18]	81.72 [78.14, 83.32]	55.82 [51.42, 59.34]	35.60 [31.73, 39.50]	26.07 [24.58, 28.83]
	**TF-IDF**	24.93 [18.39, 30.94]	**87.83 [85.85, 89.67]**	71.44 [67.02, 74.71]	**55.02 [51.12, 58.64]**	**36.97 [34.51, 39.37]**
	**SBERT**	25.43 [19.33, 32.57]	85.21 [82.91, 87.27]	68.29 [64.59, 72.87]	52.90 [47.87, 55.68]	31.68 [29.99, 33.29]
	**ColBERT**	25.07 [18.82, 31.75]	47.99 [44.02, 51.72]	**72.38 [68.91, 75.83]**	50.14 [47.32, 54.97]	32.27 [31.18, 35.97]
	**DPR**	**26.88 [19.50, 33.24]**	84.58 [82.28, 86.75]	70.46 [64.70, 73.31]	52.77 [48.79, 56.40]	28.72 [30.21, 33.71]
20-shot	**Base**	21.15 [16.82, 26.69]	82.16 [79.39, 85.28]	57.33 [53.12, 60.03]	38.89 [32.30, 45.49]	28.66 [26.78, 31.11]
	**TF-IDF**	27.36 [22.76, 33.25]	**86.49 [84.07, 88.72]**	**73.69 [70.74, 76.71]**	**58.23 [55.34, 61.24]**	**35.62 [33.27, 37.69]**
	**SBERT**	29.85 [24.48, 34.61]	86.42 [84.35, 88.38]	70.35 [66.80, 74.36]	54.78 [51.45, 57.72]	34.77 [32.98, 36.55]
	**ColBERT**	**30.40 [25.88, 36.35]**	49.11 [45.27, 53.13]	73.51 [69.41, 77.53]	53.65 [49.67, 57.83]	33.65 [30.51, 34.23]
	**DPR**	29.77 [24.34, 35.09]	84.28 [81.86, 86.91]	69.02 [65.48, 73.19]	57.42 [53.33, 61.38]	31.99 [26.95, 30.52]

Table S13. Evaluation of dynamic prompting strategies (5-shot, 10-shot, and 20-shot) using GPT-OSS across five biomedical datasets. The table presents F_1-score for each retrieval method: Base Prompt, TF-IDF, SBERT, ColBERT, and DPR, with 95% confidence intervals reported for each metric to indicate the statistical reliability of the results.

			Reddit_Impacts			*BC5CDR*			*MIMIC III*			*NCBI*			*Med-Mentions*		
			**P**	**R**	**F_1**	**P**	**R**	**F_1**	**P**	**R**	**F_1**	**P**	**R**	**F_1**	**P**	**R**	**F_1**
**5-shot**		**Base**	13.16	57.86	21.43	68.97	78.36	73.32	59.30	67.27	62.94	35.81	34.71	34.80	25.89	67.05	37.26
	**50**	**TF-IDF**	20.23	63.86	30.72	73.44	75.89	74.64	64.85	76.16	70.05	36.82	39.53	38.13	24.38	65.31	35.51
		**SBERT**	20.50	59.76	30.53	74.62	76.38	75.49	63.95	71.63	67.57	38.08	38.37	38.22	24.76	66.58	36.10
		**Avg**	20.365	61.81	**30.625**	74.03	76.135	**75.065**	64.4	73.895	**68.81**	37.45	38.95	**38.175**	24.57	65.945	**35.805**
	**100**	**TF-IDF**	18.47	56.98	27.79	75.08	77.10	76.08	67.52	76.08	71.55	47.42	49.02	48.21	24.63	68.38	36.22
		**SBERT**	21.85	64.20	32.60	77.39	78.15	77.77	65.81	74.28	69.79	49.62	49.69	49.65	25.24	65.22	36.39
		**Avg**	20.16	60.59	**30.195**	76.235	77.625	**76.925**	66.665	75.18	**70.67**	48.52	49.355	**48.93**	24.935	66.80	**36.305**
	**200**	**TF-IDF**	18.91	58.49	28.58	76.25	79.24	77.72	66.62	76.89	71.39	45.98	46.51	46.24	25.92	68.97	37.68
		**SBERT**	23.17	66.52	34.37	78.98	79.36	79.17	67.52	76.28	71.63	47.55	48.65	48.09	26.08	69.73	37.96
		**Avg**	21.04	62.505	**31.475**	77.615	79.3	**78.445**	67.07	76.585	**71.51**	46.765	47.58	**47.165**	26.00	69.35	**37.82**
	**ALL**	**TF-IDF**	18.89	58.62	28.57	78.49	81.78	80.11	66.48	74.84	70.41	48.93	50.70	49.80	26.46	72.06	38.68
		**SBERT**	23.2	66.67	34.42	77.26	83.79	80.39	64.04	72.21	67.88	50.66	49.59	50.12	26.15	68.92	37.91
		**Avg**	21.045	62.645	**31.495**	77.875	82.785	**80.25**	65.26	73.525	**69.145**	49.795	50.145	**49.96**	26.305	70.49	**38.295**
**10-shot**		**Base**	22.37	59.94	32.50	72.56	77.91	75.15	59.13	71.63	63.77	39.67	31.49	35.60	25.57	64.33	36.50
	**50**	**TF-IDF**	24.35	65.88	35.56	75.02	75.69	75.36	64.54	74.68	69.24	38.13	37.84	37.98	23.91	66.68	35.18
		**SBERT**	24.62	59.76	34.88	76.83	77.58	77.20	64.99	76.32	70.20	41.13	39.53	40.32	25.00	68.56	36.64
		**Avg**	24.485	62.82	**35.22**	75.925	76.635	**76.28**	64.765	75.5	**69.72**	39.63	38.685	**39.15**	24.455	67.62	**35.91**
	**100**	**TF-IDF**	23.24	62.73	33.92	76.55	77.76	77.15	65.73	75.56	70.31	48.47	49.22	48.85	24.59	69.05	36.27
		**SBERT**	22.09	59.13	32.16	77.75	79.49	78.61	66.01	75.44	70.41	49.19	46.90	48.02	25.46	66.90	36.88
		**Avg**	22.665	60.93	**33.04**	77.15	78.625	**77.88**	65.87	75.5	**70.36**	48.83	48.06	**48.435**	25.025	67.975	**36.575**
	**200**	**TF-IDF**	24.02	60.78	34.43	78.89	78.5	78.69	66.41	76.84	71.25	48.69	49.76	49.22	25.12	66.43	36.45
		**SBERT**	22.31	59.92	32.51	79.61	79.89	79.75	66.75	77.74	71.83	49.96	49.78	49.87	25.40	68.46	37.03
		**Avg**	23.165	60.35	**33.47**	79.25	79.195	**79.22**	66.58	77.29	**71.54**	49.325	49.77	**49.545**	25.26	67.445	**36.74**
	**ALL**	**TF-IDF**	23.53	62.65	34.21	80.82	80.32	80.57	55.79	55.34	55.56	49.59	49.41	49.50	24.03	68.00	35.51
		**SBERT**	22.27	59.76	32.45	77.72	84.94	81.17	67.67	76.09	71.63	52.84	49.94	51.35	27.61	66.88	39.08
		**Avg**	22.9	61.205	**33.33**	79.27	82.63	**80.87**	61.73	65.715	**63.595**	51.215	49.675	**50.425**	25.82	67.44	**37.295**

Table S14. Ablation results evaluating the effect of retrieval pool size on dynamic prompting using LLaMA 3-70B. Precision (P), recall (R), and F_1-score (F_1) are reported across five datasets under 5-shot and 10-shot settings. The static baseline uses randomly selected examples, while dynamic prompting employs TF-IDF and SBERT with pool sizes of 50, 100, 200, and All. Average rows reflect the mean performance of both retrievers, and best F_1 scores are highlighted.

			Reddit_Impacts	*BC5CDR*	*MIMIC III*	*NCBI*	*Med-Mentions*
**5-shot**		**Base**	21.43 [14.24, 28.80]	73.32 [72.27, 74.26]	62.94 [57.07, 65.79]	34.80 [28.57, 35.44]	37.26 [35.45, 39.08]
	**50**	**TF-IDF**	30.72 [23.93, 37.97]	74.64 [71.67, 77.38]	70.05 [65.87, 73.60]	38.13 [32.89, 40.50]	35.51 [32.64, 39.90]
		**SBERT**	30.53 [23.43, 37.76]	75.49 [72.39, 78.37]	67.57 [63.66, 71.46]	38.22 [32.47, 39.20]	36.10 [34.67, 40.86]
		**Avg**	**30.625**	**75.065**	**68.81**	**38.175**	**35.805**
	**100**	**TF-IDF**	27.79 [20.46, 34.32]	76.08 [72.75, 79.36]	71.55 [67.84, 74.82]	48.21 [42.18, 50.34]	36.22 [34.68, 40.56]
		**SBERT**	32.60 [25.43, 39.85]	77.77 [75.16, 80.56]	69.79 [66.04, 73.20]	49.65 [42.61, 51.61]	36.39 [34.62, 40.84]
		**Avg**	**30.195**	**76.925**	**70.67**	**48.93**	**36.305**
	**200**	**TF-IDF**	28.58 [21.86, 35.71]	77.72 [74.56, 80.44]	71.39 [67.65, 75.20]	46.24 [40.00, 49.79]	37.68 [35.97, 41.78]
		**SBERT**	34.37 [27.42, 41.09]	79.17 [76.54, 82.41]	71.63 [68.03, 74.85]	48.09 [42.57, 50.00]	37.96 [34.87, 41.80]
		**Avg**	**31.475**	**78.445**	**71.51**	**47.165**	**37.82**
	**ALL**	**TF-IDF**	28.57 [21.74, 36.06]	80.11 [79.25, 81.00]	70.41 [66.87, 73.76]	49.80 [46.38, 53.03]	38.68 [35.67, 40.81]
		**SBERT**	34.42 [26.28, 41.52]	80.39 [79.50, 81.33]	67.88 [64.09, 71.69]	50.12 [46.89, 53.66]	37.91 [36.02, 39.81]
		**Avg**	**31.495**	**80.25**	**69.145**	**49.96**	**38.295**
**10-shot**		**Base**	32.50 [26.94, 42.26]	75.15 [74.65, 76.67]	63.77 [58.59, 67.75]	35.60 [32.17, 39.12]	36.50 [35.73, 39.57]
	**50**	**TF-IDF**	35.56 [27.96, 43.06]	75.36 [72.09, 78.19]	69.24 [65.70, 72.76]	37.98 [32.62, 40.11]	35.18 [33.50, 40.88]
		**SBERT**	34.88 [27.85, 42.44]	77.20 [74.12, 80.34]	70.20 [66.61, 73.72]	40.32 [36.37, 44.27]	36.64 [34.56, 41.10]
		**Avg**	**35.22**	**76.28**	**69.72**	**39.15**	**35.91**
	**100**	**TF-IDF**	33.92 [26.21, 41.60]	77.15 [74.40, 79.92]	70.31 [66.39, 73.84]	48.85 [43.01, 51.08]	36.27 [34.31, 40.84]
		**SBERT**	32.16 [25.04, 39.22]	78.61 [75.67, 81.83]	70.41 [66.71, 73.89]	48.02 [45.33, 52.05]	36.88
		**Avg**	**33.04**	**77.88**	**70.36**	**48.435**	**36.575**
	**200**	**TF-IDF**	34.43 [27.26, 41.47]	78.69 [75.81, 82.28]	71.25 [67.10, 74.63]	49.22 [43.98, 52.20]	36.45 [34.16, 40.24]
		**SBERT**	32.51 [25.35, 39.68]	79.75 [76.77, 82.93]	71.83 [67.62, 74.75]	49.87 [42.41, 51.89]	37.03 [35.49, 41.81]
		**Avg**	**33.47**	**79.22**	**71.54**	**49.545**	**36.74**
	**ALL**	**TF-IDF**	34.21 [27.24, 42.03]	80.57 [79.65, 81.47]	55.56 [53.11, 60.44]	49.50 [46.05, 52.92]	35.51 [34.75, 37.45]
		**SBERT**	32.45 [25.33, 39.63]	81.17 [80.26, 82.03]	71.63 [67.75, 75.15]	51.35 [47.49, 55.16]	39.08 [36.39, 41.38]
		**Avg**	**33.33**	**80.87**	**63.595**	**50.425**	**37.295**

Table S15. Evaluation of retrieval-based ablation settings (5-shot and 10-shot) using two retrieval engines, TF-IDF and SBERT, using Llama 3 across five datasets. The table presents F_1-score with 95% confidence intervals for each retrieval pool size (50, 100, 200, and ALL), alongside the "Base" setting, which does not use retrieval. Average rows summarize the mean performance of the two retrieval engines under each configuration to illustrate the effect of retrieval size on dynamic prompting performance.

C Detailed Task-specific Static Prompts

Prompt Strategies	*Reddit-Impacts*
Basic Prompt	**[Task Description]:** You are a medical AI trained to identify and classify tokens into three categories: Clinical Impacts, Social Impacts, and Outside ('O'). Your task is to extract and classify the clinical and social impacts from this dataset, considering your knowledge of the lifestyle of this population and the potential clinical and social impacts they might experience.
	**[Entity Types with Definitions]:** 'Clinical Impacts' refer to tokens describing the effects, consequences, or impacts of substance use on individual health or well-being, as defined in UMLS. 'Social Impacts' describe the societal, interpersonal, or community-level effects, also based on UMLS definitions. Any token not falling into these categories should be labeled as 'O'.
	**[Format Specification]:** For example, the sentence 'I was a codeine addict.' is tokenized and labeled as follows: ['I', 'was', 'a', 'codeine', 'addict', '.'] with labels ['O', 'O', 'O', 'Clinical Impacts', 'Clinical Impacts', 'O']. Your task is to predict and return the label for each provided token, ensuring the number of output labels matches the number of input tokens exactly. The output format should be tokens with their labels: ['I-O', 'was-O', 'a-O', 'codeine-Clinical Impacts', 'addict-Clinical Impacts', '.-O'].
Description of datasets	The data you are working with has been collected from 14 forums on Reddit (subreddits) that focused on prescription and illicit opioids, and medications for opioid use disorder. This dataset represents a social media context, coming from individuals who may use prescription and illicit opioids and stimulants.
High-frequency instances	In this dataset, high-frequency clinical impacts include 'withdrawal', 'rehab', 'addicted', 'detox', 'overdosed', and 'rehabs'. High-frequency social impacts include 'lost', 'homeless', 'charged', 'streets', 'jail', and 'disorderly'.
UMLS knowledge	The Unified Medical Language System (UMLS) is developed by the U.S. National Library of Medicine (NLM) to integrate and standardize diverse medical terminologies and coding systems. It consists of three main components: the Metathesaurus, Semantic Network, and SPECIALIST Lexicon, supporting medical information retrieval and semantic analysis. You understand medical terminology and concepts from UMLS.
Error analysis	Possible analysis of prediction errors: If a sentence describes the background information of an event, facility, or project, then even if it mentions keywords related to social impact like 'at jail', it still cannot be determined as describing a patient being in jail. It is essential to clearly determine whether the sentence is describing the patient's condition. Second, if the sentence is about the usage, operation, or introduction of a drug or medicine, it does not belong to the patient's clinical impacts, even if it mentions some symptoms. Pay attention to whether the sentence contains words like 'if' that indicate conditions.

Table S16. Specific static prompts for each component we used for the REDDIT-IMPACTS dataset.

Prompt Strategies	*BC5CDR*
Basic Prompt	**[Task Description]:** You are a medical AI trained to identify and classify tokens into three categories: 'Disease', 'Chemical' and Outside ('O'). Your task is to extract and classify the Disease and Chemical related concepts from this dataset.
	**[Entity Types with Definitions]:** 'Disease' is a particular abnormal condition that adversely affects the structure or function of all or part of an organism and is not immediately due to any external injury. Diseases are often known to be medical conditions that are associated with specific signs and symptoms. A disease may be caused by external factors such as pathogens or by internal dysfunctions. For example, internal dysfunctions of the immune system can produce a variety of different diseases, including various forms of immunodeficiency, hypersensitivity, allergies, and autoimmune disorders. 'Chemical' in this context refers to substances or compounds with specific chemical properties and structures. These can include drugs, neurotransmitters, elements or ions, vitamins, and other medically relevant chemicals. Any token not falling into Disease categories should be labeled as 'O'.
	**[Format Specification]:** For example, the sentence 'The hypotensive effect of 100 mg / kg alpha-methyldopa was also partially reversed by naloxone .' is tokenized and labeled as follows: ['The', 'hypotensive', 'effect', 'of', '100', 'mg', '/', 'kg', 'alpha-methyldopa', 'was', 'also', 'partially', 'reversed', 'by', 'naloxone', '.'] with labels ['O', 'Disease', 'O', 'O', 'O', 'O', 'O', 'O', 'Chemical', 'O', 'O', 'O', 'O', 'O', 'Chemical', 'O']. Your task is to predict and return the label for each provided token, ensuring the number of output labels matches the number of input tokens exactly. The output format should include tokens with their labels: ['The-O', 'hypotensive-Disease', 'effect-O', 'of-O', '100-O', 'mg-O', '/-O', 'kg-O', 'alpha-methyldopa-Chemical', 'was-O', 'also-O', 'partially-O', 'reversed-O', 'by-O', 'naloxone-Chemical', '.-O'].
Description of datasets	The data you are working with is BC5CDR dataset, a benchmark dataset for biomedical natural language processing, created from PubMed abstracts. It includes annotations for two entity types—chemicals and diseases—and their relationships, specifically chemical-induced disease interactions. The dataset is widely used for tasks such as named entity recognition and relation extraction, supporting research in biomedical text mining and information extraction.
High-frequency instances	In this dataset, high frequency of 'Disease' include 'pain', 'toxicity', 'renal', 'failure', 'disease', 'hypotension'; high frequency of 'Chemical' include 'cocaine', 'acid', 'dopamine', 'nicotine', 'morphine', 'lithium'.
UMLS knowledge	The Unified Medical Language System (UMLS) is developed by the U.S. National Library of Medicine (NLM) to integrate and standardize diverse medical terminologies and coding systems. It consists of three main components: the Metathesaurus, Semantic Network, and SPECIALIST Lexicon, supporting medical information retrieval and semantic analysis. You understand medical terminology and concepts from UMLS.
Error analysis	Possible analysis of prediction errors: The prediction errors mainly stem from challenges in distinguishing between entity boundaries and contextual usage. For instance, multi-token entities were partially labeled, causing boundary mismatches. Additionally, certain terms such as "receptor" or "antagonist" were incorrectly labeled as 'O,' despite being part of chemical-related entities. Misclassification also occurred in sentences with conditional phrases or background information, where the relation between entities was not accurately captured. Furthermore, entities mentioned in descriptive or abstract contexts, were sometimes overlooked. These errors highlight difficulties in handling complex sentence structures, context-specific classification, and multi-token entity recognition.

Table S17. Specific static prompts for each component we used for the BC5CDR dataset.

Prompt Strategies	*MIMIC III*
Basic Prompt	**[Task Description]:** You are a medical AI trained to identify and classify tokens into 13 categories: 'CONDITION/SYMPTOM', 'DRUG', 'AMOUNT', 'TIME', 'MEASUREMENT', 'LOCATION', 'EVENT', 'FREQUENCY', 'ORGANIZATION', 'DATE', 'AGE', 'GENDER' and Outside ('O'). Your task is to extract and classify the concepts from this dataset.<br>**[Entity Types with Definitions]:** 'ORGANIZATION' refers to entities or groups associated with healthcare or emergency medical services. These could be specific departments, teams, or services within a medical or emergency response organization. 'DATE' in this context refers to specific calendar dates. These dates are typically used to mark particular events, appointments, or deadlines. 'AGE' in this context refers to the length of time that a person has lived or the number of years since their birth. It can be expressed in various formats, including numerical values, abbreviated forms, or written out in words. 'GENDER' in this context refers to the socially constructed roles, behaviors, activities, and attributes that a given society considers appropriate for men and women. It encompasses the identities of 'male' and 'female,' which are often associated with biological sex but are also shaped by cultural and social factors. 'FREQUENCY' in this context refers to the rate or regularity at which an event or phenomenon occurs. It can describe how often something happens, ranging from sporadic or irregular occurrences to more regular or constant patterns. 'EVENT' in this context refers to specific occurrences or actions that take place, particularly in a medical or clinical setting. These can include procedures, assessments, or other significant incidents. 'LOCATION' in this context refers to specific places or areas, particularly within a healthcare or medical setting. These can include types of facilities, specific locations within a facility, or other relevant places. 'MEASUREMENT' in this context refers to quantitative assessments or values used to evaluate specific physiological or medical parameters. These can include vital signs, laboratory test results, numerical values, or other metrics related to patient health. 'TIME' in this context refers to specific points or periods in the temporal continuum, particularly as they relate to healthcare or medical events. These can include general time references, specific durations, or events tied to time. 'AMOUNT' in this context refers to specific quantities or dosages, particularly in a medical or pharmaceutical setting. These can include measurements of medication, frequency or number of administrations, and methods of delivery. 'DRUG' in this context refers to specific medications or pharmaceutical substances used in the treatment, prevention, or diagnosis of diseases. These can include brand names, generic names, or forms of administration. 'CONDITION/SYMPTOM' in this context refers to physical or subjective signs that indicate a medical condition or disease. These can include sensations of discomfort, specific types of pain or discomfort, respiratory issues, or gastrointestinal symptoms. Any token not falling into categories above should be labeled as 'O'.<br>**[Format Specification]:** For example, the sentence 'The patient was readmitted to the hospital on 2195-6-6 due to fevers to 103 at the rehabilitation facility despite being on intravenous antibiotics HISTORY OF PRESENT ILLNESS 55 year-old female presents with 2/5 week history of non-bloody diarrhea' is tokenized and labeled as follows: ['The', 'patient', 'was', 'readmitted', 'to', 'the', 'hospital', 'on', '2195-6-6', 'due', 'to', 'fevers', 'to', '103', 'at', 'the', 'rehabilitation', 'facility', 'despite', 'being', 'on', 'intravenous', 'antibiotics', 'HISTORY', 'OF', 'PRESENT', 'ILLNESS', '55', 'year-old', 'female', 'presents', 'with', '2/5', 'week', 'history', 'of', 'non-bloody', 'diarrhea'] with labels ['O', 'O', 'O', 'EVENT', 'O', 'LOCATION', 'LOCATION', 'O', 'O', 'O', 'O', 'MEASUREMENT', 'MEASUREMENT', 'MEASUREMENT', 'O', 'LOCATION', 'LOCATION', 'LOCATION', 'O', 'O', 'O', 'DRUG', 'DRUG', 'O', 'O', 'O', 'O', 'AGE', 'O', 'GENDER', 'O', 'O', 'AMOUNT', 'AMOUNT', 'O', 'O', 'CONDITION/SYMPTOM', 'CONDITION/SYMPTOM']. Your task is to predict and return the label for each provided token, ensuring the number of output labels matches the number of input tokens exactly. The output format should include tokens with their labels: ['The-O', 'patient-O', 'was-O', 'readmitted-EVENT', 'to-O', 'the-LOCATION', 'hospital-LOCATION', 'on-O', '2195-6-6-O', 'due-O', 'to-O', 'fevers-MEASUREMENT', 'to-MEASUREMENT', '103-MEASUREMENT', 'at-O', 'the-LOCATION', 'rehabilitation-LOCATION', 'facility-LOCATION', 'despite-O', 'being-O', 'on-O', 'intravenous-DRUG', 'antibiotics-DRUG', 'HISTORY-O', 'OF-O', 'PRESENT-O', 'ILLNESS-O', '55-AGE', 'year-old-O', 'female-GENDER', 'presents-O', 'with-O', '2/5-AMOUNT', 'week-AMOUNT', 'history-O', 'of-O', 'non-bloody-CONDITION/SYMPTOM', 'diarrhea-CONDITION/SYMPTOM'].
Description of datasets	The data you are working with is MIMIC-III (Medical Information Mart for Intensive Care) dataset, a large, publicly available database containing de-identified health data from critical care patients at the Beth Israel Deaconess Medical Center. It includes structured data, such as demographics, lab results, and vital signs, as well as unstructured data, such as clinical notes and discharge summaries. The dataset is widely used for research in machine learning, natural language processing, and clinical decision support to improve healthcare outcomes.
High-frequency instances	In this dataset, high frequency of 'CONDITION/SYMPTOM' include 'pain', 'chest', 'cough', 'breath', 'nausea', 'abdominal'; high frequency of 'DRUG' include 'iv', 'lasix', 'ceftriaxone', 'oxygen', 'ns', 'coumadin'; high frequency of 'AMOUNT' include 'iv', '2', '1', 'mg', 'days', 'one'; high frequency of 'TIME' include 'day', 'admission', 'prior', 'last', 'ago', 'morning'; high frequency of 'MEASUREMENT' include 'bp', 'hr', 'pressure', 'blood', 'rr', 'rate', 'heart'; high frequency of 'LOCATION' include 'hospital', 'right', 'home', 'floor', 'emergency', 'micu'; high frequency of 'EVENT' include 'ct', 'placed', 'cxr', 'intubated', 'exam', 'review'; high frequency of 'FREQUENCY' include 'chronic', 'intermittent', 'daily', 'occasionally', 'frequent', 'intermittently'; high frequency of 'ORGANIZATION' include 'ems', 'service', 'surgery', 'pcp', 'emergency', 'neuro', 'medicine'; high frequency of 'DATE' include '2171114', '21491117'; high frequency of 'AGE' include '60', '80yo', '78', '61', 'seventyeightyearold', '69'; high frequency of 'GENDER' include 'man', 'woman', 'f', 'male', 'female', 'm'.
UMLS knowledge	The Unified Medical Language System (UMLS) is developed by the U.S. National Library of Medicine (NLM) to integrate and standardize diverse medical terminologies and coding systems. It consists of three main components: the Metathesaurus, Semantic Network, and SPECIALIST Lexicon, supporting medical information retrieval and semantic analysis. You understand medical terminology and concepts from UMLS.
Error analysis	The prediction errors stem from several factors. Entity boundary recognition issues were common, particularly with multi-token entities like "shortness of breath" or "paroxysmal nocturnal dyspnea," where some tokens were missed or incorrectly labeled as 'O.' Additionally, the model struggled with entity type confusion, such as distinguishing "pain" as a symptom versus its contextual use related to location. Context-dependent misinterpretations also contributed to errors, especially in handling negations like "denies chest pain" or temporal references such as "last few months." Overlapping entities posed further challenges, where closely related terms (e.g., "MI" and "CABG") interfered with accurate classification. Finally, rare or unseen entities in the training data led to occasional misclassifications, highlighting gaps in the model's ability to generalize.

Table S18. Specific static prompts for each component we used for the MIMIC III dataset.

Prompt Strategies	*NCBI*
Basic Prompt	**[Task Description]:** You are a medical AI trained to identify and classify tokens into five categories: DiseaseClass, SpecificDisease, Modifier, CompositeMention and Outside ('O'). Your task is to extract and classify the DiseaseClass, SpecificDisease, Modifier and CompositeMention from this dataset.
	**[Entity Types with Definitions]:** 'DiseaseClass' refers to a classification system or category used to group various medical conditions or diseases based on certain characteristics, such as their nature, affected biological systems, or underlying causes. 'SpecificDisease' appears to describe particular diseases that are identified and classified based on their specific clinical features, genetic origins, or biochemical abnormalities. 'Modifier' refers to specific attributes or variations or conditions that can modify or influence the presentation, progression, or characteristics of a disease, alter the manifestation or course of a disease, potentially affecting its diagnosis, treatment, and prognosis. 'CompositeMention' describes medical conditions or characteristics that are composed of several elements or features, often involving multiple tissues, organs, or systems. Any token not falling into these categories should be labeled as 'O'.
	**[Format Specification]:** For example, the sentence 'Histidinemia. Classical and atypical form in siblings.' is tokenized and labeled as follows: ['Histidinemia.', 'Classical', 'and', 'atypical', 'form', 'in', 'siblings.'] with labels ['SpecificDisease', 'O', 'O', 'O', 'O', 'O', 'O']. Your task is to predict and return the label for each provided token, ensuring the number of output labels matches the number of input tokens exactly. The output format should include tokens with their labels: ['Histidinemia.-SpecificDisease', 'Classical-O', 'and-O', 'atypical-O', 'form-O', 'in-O', 'siblings.-O'].
Description of datasets	The data you are working with is NCBI disease corpus, a collection of 793 PubMed abstracts fully annotated at the mention and concept level to serve as a research resource for the biomedical natural language processing community. Each PubMed abstract was manually annotated by two annotators with disease mentions and their corresponding concepts in Medical Subject Headings (MeSH) or Online Mendelian Inheritance in Man (OMIM). The public release of the NCBI disease corpus contains 6892 disease mentions, which are mapped to 790 unique disease concepts. Of these, 88 percent link to a MeSH identifier, while the rest contain an OMIM identifier. We were able to link 91 percent of the mentions to a single disease concept, while the rest are described as a combination of concepts.
High-frequency instances	In this dataset, high-frequency 'DiseaseClass' include 'disorder', 'abnormalities', 'tumors', 'mental', 'disorders', 'retardation'. High-frequency 'SpecificDisease' include 'deficiency', 'syndrome', 'dystrophy', 'familial', 'myotonic', 'colorectal'. High-frequency 'Modifier' include 'tumor', 'tumour', 'APC', 'choroideremia', 'DM', 'DMD'. High-frequency 'CompositeMention' include 'breast', 'ovarian', 'cancer', 'muscular', 'andor', 'becker'.
UMLS knowledge	The Unified Medical Language System (UMLS) is developed by the U.S. National Library of Medicine (NLM) to integrate and standardize diverse medical terminologies and coding systems. It consists of three main components: the Metathesaurus, Semantic Network, and SPECIALIST Lexicon, supporting medical information retrieval and semantic analysis. You understand medical terminology and concepts from UMLS.
Error analysis	The prediction errors in the NCBI dataset primarily stem from challenges in distinguishing composite mentions and modifiers within complex biomedical contexts. For instance, entities like "BRCA1 gene" were incorrectly segmented, with "BRCA1" labeled as a modifier instead of being part of the composite mention. Additionally, multi-token composite mentions such as "breast and ovarian cancer" were not consistently labeled, with individual tokens occasionally missed or misclassified. Contextual ambiguity, such as distinguishing between mentions of general biological terms (e.g., "tumor") and their specific functional roles (e.g., "tumor suppressor"), also contributed to errors.

Table S19. Specific static prompts for each component we used for the NCBI dataset.

Prompt Strategies	*Med-Mentions*
Basic Prompt	**[Task Description]:** You are a medical AI trained to identify and classify tokens into two categories: Disease and Outside ('O'). Your task is to extract and classify the Disease related concepts from this dataset.
	**[Entity Types with Definitions]:** 'Disease' is a particular abnormal condition that adversely affects the structure or function of all or part of an organism and is not immediately due to any external injury. Diseases are often known to be medical conditions that are associated with specific signs and symptoms. A disease may be caused by external factors such as pathogens or by internal dysfunctions. For example, internal dysfunctions of the immune system can produce a variety of different diseases, including various forms of immunodeficiency, hypersensitivity, allergies, and autoimmune disorders. Any token not falling into Disease categories should be labeled as 'O'.
	**[Format Specification]:** For example, the sentence 'A total of 200 children and adolescents with type 1 diabetes, ages 9-18 years, completed the DEPS-R Turkish version.' is tokenized and labeled as follows: ['A', 'total', 'of', '200', 'children', 'and', 'adolescents', 'with', 'type', '1', 'diabetes,', 'ages', '9-18', 'years,', 'completed', 'the', 'DEPS-R', 'Turkish', 'version.'] with labels ['O', 'O', 'O', 'O', 'Disease', 'O', 'Disease', 'O', 'Disease', 'Disease', 'Disease', 'Disease', 'O', 'Disease', 'O', 'O', 'Disease', 'Disease', 'Disease']. The output format should include tokens with their labels: ['A-O', 'total-O', 'of-O', '200-O', 'children-Disease', 'and-O', 'adolescents-Disease', 'with-O', 'type-Disease', '1-Disease', 'diabetes,-Disease', 'ages-Disease', '9-18-O', 'years,-Disease', 'completed-O', 'the-O', 'DEPS-R-Disease', 'Turkish-Disease', 'version.-Disease'].
Description of datasets	The data you are working with is Med-Mentions, a new manually annotated resource for the recognition of biomedical concepts. What distinguishes Med-Mentions from other annotated biomedical corpora is its size (over 4,000 abstracts and over 350,000 linked mentions), as well as the size of the concept ontology (over 3 million concepts from UMLS 2017) and its broad coverage of biomedical disciplines.
High-frequency instances	In this dataset, high frequency 'Disease' related entities include 'patients', 'cells', 'treatment', 'cancer', 'analysis', 'disease', 'clinical'.
UMLS knowledge	The Unified Medical Language System (UMLS) is developed by the U.S. National Library of Medicine (NLM) to integrate and standardize diverse medical terminologies and coding systems. It consists of three main components: the Metathesaurus, Semantic Network, and SPECIALIST Lexicon, supporting medical information retrieval and semantic analysis. You understand medical terminology and concepts from UMLS.
Error analysis	The prediction errors in the Med-Mentions dataset are primarily due to challenges in identifying complex and overlapping disease mentions, as well as distinguishing between general biomedical terms and specific disease entities. Multi-token entities such as "renal pedicle occlusion" or "intention-to-treat analyses" were often partially labeled, with some tokens being misclassified or excluded. Additionally, the presence of nested or overlapping mentions, such as "prostate cancer" and its relationship to broader contexts like "treatment disparities," led to inconsistent labeling. The model also struggled with domain-specific terminology, misclassifying general terms like "maternal genotype" or "outcome" as disease mentions. These errors highlight limitations in handling nuanced biomedical language, especially when entities span multiple tokens or overlap with related terms.

Table S20. Specific static prompts for each component we used for the Med-Mentions dataset.
